# Striatal parvalbumin interneurons are activated in a mouse model of cerebellar dystonia

**DOI:** 10.1242/dmm.050338

**Published:** 2024-05-14

**Authors:** Taku Matsuda, Ryoma Morigaki, Hiroaki Hayasawa, Hiroshi Koyama, Teruo Oda, Kazuhisa Miyake, Yasushi Takagi

**Affiliations:** ^1^ Department of Neurosurgery, Graduate School of Biomedical Sciences, Tokushima University, Tokushima 770-8503, Japan; ^2^ Department of Advanced Brain Research, Graduate School of Biomedical Sciences, Tokushima University, Tokushima 770-8503, Japan; ^3^ Parkinson's Disease and Dystonia Research Center, Tokushima University Hospital, Tokushima 770-8503, Japan

**Keywords:** Cerebellar dystonia, Parvalbumin interneuron, Cholinergic interneuron, Dopamine D1 receptor agonist, Dopamine D2 receptor antagonist

## Abstract

Dystonia is thought to arise from abnormalities in the motor loop of the basal ganglia; however, there is an ongoing debate regarding cerebellar involvement. We adopted an established cerebellar dystonia mouse model by injecting ouabain to examine the contribution of the cerebellum. Initially, we examined whether the entopeduncular nucleus (EPN), substantia nigra pars reticulata (SNr), globus pallidus externus (GPe) and striatal neurons were activated in the model. Next, we examined whether administration of a dopamine D1 receptor agonist and dopamine D2 receptor antagonist or selective ablation of striatal parvalbumin (PV, encoded by *Pvalb*)-expressing interneurons could modulate the involuntary movements of the mice. The cerebellar dystonia mice had a higher number of cells positive for c-fos (encoded by *Fos*) in the EPN, SNr and GPe, as well as a higher positive ratio of c-fos in striatal PV interneurons, than those in control mice. Furthermore, systemic administration of combined D1 receptor agonist and D2 receptor antagonist and selective ablation of striatal PV interneurons relieved the involuntary movements of the mice. Abnormalities in the motor loop of the basal ganglia could be crucially involved in cerebellar dystonia, and modulating PV interneurons might provide a novel treatment strategy.

## Teaser

**Summary:** In a mouse model of cerebellar dystonia, parvalbumin interneurons were activated and the basal ganglia circuitry was altered, indicating the involvement of the cortico-basal ganglia–thalamo-cortical circuit in cerebellar dystonia.

## INTRODUCTION

Dystonia is a movement disorder characterized by twisting, repetitive or patterned movements as well as abnormal postures induced by sustained involuntary muscle contractions (Albanese et al., 2013; Kaji et al., 2018; Vidailhet et al., 2005). Dysfunction of the motor loop, specifically the cortico-basal ganglia–thalamo-cortical loop, is considered to be involved in the pathogenesis of dystonia (Breakefield et al., 2008; Kaji et al., 2018; Morigaki and Miyamoto, 2022). Currently, deep brain stimulation (DBS) of the motor loop structures is among the main remedies for medication-refractory dystonia (Fan et al., 2021; Lin et al., 2019; Morigaki and Miyamoto, 2022; Ozturk et al., 2021; Vidailhet et al., 2005). However, evidence supporting the hypothesis that the cerebellum could be involved in dystonia is continuously emerging (Bologna and Berardelli, 2018; Bostan and Strick, 2018; Filip et al., 2013; Kaji et al., 2018; Morigaki et al., 2021; Prudente et al., 2014; Shakkottai et al., 2017; Shakkottai, 2014; Tewari et al., 2017). The cerebellum plays an important role in controlling movements, such as coordination, balance and motor skills; ataxia is the most general symptom of their dysfunction (Beckinghausen and Sillitoe, 2019). Morphologically, abnormal cerebellar gray matter in patients with dystonia has been reported (Delmaire et al., 2007; Draganski et al., 2003; Obermann et al., 2007). Furthermore, the abnormal cerebello-thalamo-striatal connection has been reported in patients with dystonia via diffusion tensor imaging, functional magnetic resonance imaging and positron emission tomography (Lehéricy et al., 2013; Reeber et al., 2013; Nieuwhof et al., 2022). Recently, it has been reported that DBS of the cerebellum alleviates dystonia (Coenen et al., 2011; França et al., 2018; Horisawa et al., 2019, 2021). These findings suggest that the dysfunction of large regions of the brain network, involving the basal ganglia and cerebellum, causes dystonia, and dystonia due to cerebellar dysfunction is known as ‘cerebellar dystonia’. The precise pathophysiology of cerebellar dystonia remains unclear. Prudente et al. (2014) proposed that suppression of the cerebellum could generate ataxia; however, cerebellar activation causes dystonia. Shakkottai et al. (2017) hypothesized that ataxia and dystonia might be the same spectrum disorder. The manifestation of ataxia and dystonia might be determined based on the degree of irregularity of cerebellar output.

Several kinds of genetic cerebellar dystonia mouse models have been reported. LeDoux et al. (1993) first reported abnormal cerebellar output in genetically dystonic rats. Campbell and Hess (1998) reported cerebellar circuit activation in the tottering mutant mouse, the generalized dystonic movements of which were caused by a mutation in the gene *Cacna1a*, encoding the α-subunit of the Cav2.1 calcium channel. Additionally, a few cerebellar dystonia mouse models were established through drug injection into the cerebellum. Pizoli et al. (2002) established a mouse model of cerebellar dystonia by injecting kainite into the cerebellum. Calderon et al. (2011) established a mouse model of cerebellar dystonia through chronic infusion of ouabain, an Na^+^/K^+^ ATPase inhibitor, into the cerebellum. Genetic models can duplicate human diseases and are good for understanding their mechanism, although they are complicated regarding nervous system diseases. The drug-induced dystonia model mouse is easy and useful for establishing dystonia pathophysiologically, although these mice do not exactly reflect patient conditions (Hess and Jinnah, 2015). In this study, we used Calderon's model (Calderon et al., 2011) to examine the influence of cerebellar abnormalities on basal ganglia circuitry and investigate its pathophysiology.

In cerebellar dystonia, the elevated cerebellar output activity affects the cerebello-thalamo-striatal circuit and may cause dystonia (Wilson and Hess, 2013). Fremont et al. (2014) reported high-frequency burst firing of the deep cerebellar nucleus (DCN), and Chen et al. (2014) found that dystonic postures were related to their abnormal high-frequency burst firing in the dorsal striatum of the cerebellar dystonia mouse model induced by chronic infusion of ouabain, although their origin was not specified. White and Sillitoe (2017) reported that aberrant activity of the DCN was found in dystonic mice, generated through genetic silencing of the olivocerebellar synapse. Neychev et al. (2008) revealed the disruption of the cerebellar–basal ganglia connection in a mouse model of cerebellar dystonia. Although these reports suggest that abnormal changes in cerebellar outputs are key to the pathogenesis of dystonia, not enough evidence exists regarding how they affect the striatum; therefore, more research is expected on this disease.

The striatum is the largest nucleus of the cortico-basal ganglia–thalamo-cortical motor loop. Medium spiny neurons (MSNs) constitute 95% of the neurons in the striatum, whereas interneurons such as cholinergic and parvalbumin (PV, encoded by *Pvalb*)-expressing interneurons (hereafter PV interneurons) constitute the remaining 5% (Capetian et al., 2014). There are two pathways in motor control: direct and indirect pathways. Activation of the direct pathway leads to the inhibition of the substantia nigra pars reticulata (SNr) and globus pallidus internus (GPi), followed by the activation of the thalamus and cortex and induction of locomotor movements. However, activation of the indirect pathway leads to the activation of the GPi/SNr by suppression of the globus pallidus externus (GPe) and activation of the subthalamic nucleus (STN), followed by the suppression of the thalamus and cortex, which reduces movements (Calabresi et al., 2014). The interneurons regulate MSNs and could be crucially involved in generating movement disorders, including dystonia (Gandham et al., 2020; Pisani et al., 2007; Scarduzio et al., 2017; Tassone et al., 2021), Huntington's disease (Crevier-Sorbo et al., 2020; Holley et al., 2019; Morigaki and Goto, 2017; Pisani et al., 2007; Rallapalle et al., 2021) and Tourette syndrome (Abudukeyoumu et al., 2019; Kalanithi et al., 2005; Rapanelli et al., 2017). Specifically, cholinergic interneurons are hyperactivated in dystonia; furthermore, anti-cholinergic drugs are frequently used to relax muscle tonus (Jankovic, 2013; Scarduzio et al., 2017; Tassone et al., 2021). The correlation between these interneurons and cerebellar dystonia remains unclear, although the abnormal cerebellar activity may induce the dysfunction of striatal interneurons (Morigaki et al., 2021). Involuntary movements generated by cerebellar dystonia are frequently refractory by drug or surgical therapy and severely impair daily activities; thus, the establishment of new therapy is crucial. Therefore, this study aimed to examine the relationship between cerebellar dystonia and basal ganglia circuitry, which could inform the establishment of effective treatments.

## RESULTS

### Establishment of the mouse model of cerebellar dystonia and behavioral analysis

**Fig. 1.**
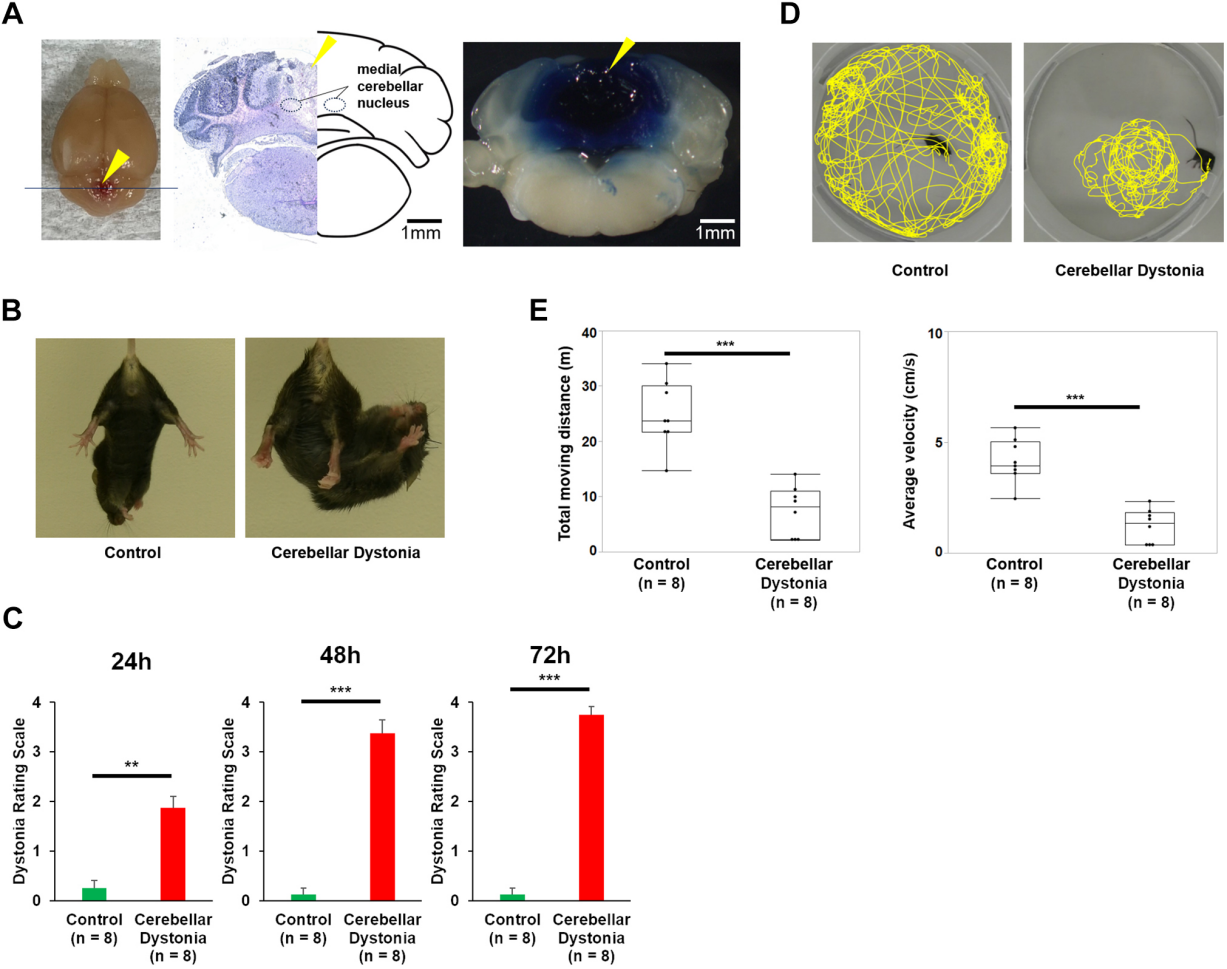
**Comparisons between cerebellar dystonia and control mice.** (A) Extracted brain of the mouse model of cerebellar dystonia (left), Hematoxylin and Eosin staining of the section and its illustration (middle), and the section dyed by 0.5% Evans Blue instead of ouabain (right). The blue line in the left panel indicates the cutline of the sections, and yellow arrowheads show the tract of the inserted cannula. (B) The control and cerebellar dystonia mice hung by their tails at 24 h post operation. (C) Postoperative changes in the scores of the dystonia rating scale for the control and cerebellar dystonia mice (*n*=8 in each group). Bars represent the mean±s.e.m. (D) Tracing of the open-field tests for the control and cerebellar dystonia mice. (E) Comparisons of the total moving distance and average velocity between the control and cerebellar dystonia mice (*n*=8 in each group). Box plots show the interquartile range, whiskers show the minimum and maximum values, and the median is marked with a line. **P<0.01; ***P<0.001; Mann–Whitney *U*-test.

Behavioral analysis of control and cerebellar dystonia mice was performed. An extracted brain of the mouse model of cerebellar dystonia and the distribution of the drugs are shown in Fig. 1A. At 24 h post operation, we observed dystonic postures of the limbs and trunk distortion in the cerebellar dystonia mice but not in the control mice (Fig. 1B). The severity of dystonia worsened over time, and the condition progressed into severe generalized dystonia after 72 h post operation (Movie 1). At 72 h post operation, the dystonia rating scale scores were higher in the cerebellar dystonia mice than in the control mice (r=0.89, *P*=0.0004, Mann–Whitney *U-*test) (Fig. 1C). The cerebellar dystonia mice showed a significantly shorter total moving distance than the control mice, and their average total velocity was slower (r=0.83, *P*=0.0009 and r=0.83, *P*=0.0009, respectively; Mann–Whitney *U-*test, respectively) (Fig. 1D,E). These results suggested impairments in both the accuracy and degree of movements.

### PV interneurons were activated in the striatum of the cerebellar dystonia mice

**Fig. 2.**
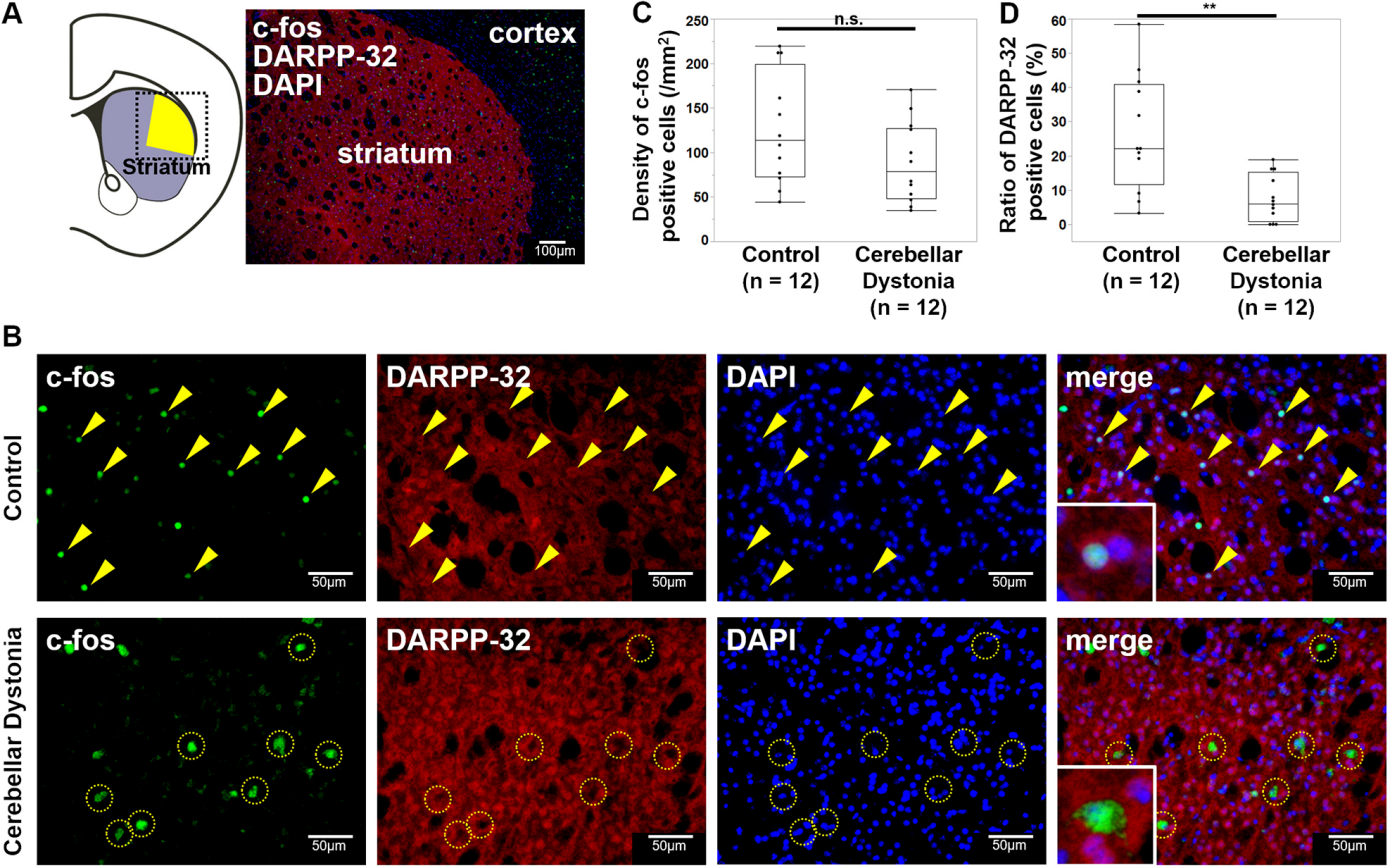
**Immunohistochemistry of the striatum in control and cerebellar dystonia mice.** (A) Schema of the brain slice and immunostaining of the striatum using antibodies against c-fos (green) and DARPP-32 (red) and staining with DAPI (blue) in the square. The yellow area shows the dorsolateral striatum, which was determined according to a previous report (Morigaki et al., 2020). (B) Immunostaining of c-fos (green) and DARPP-32 (red) in the striatum. The yellow arrowheads show c-fos-positive and DARPP-32-positive cells. The yellow dotted circles show c-fos-positive and DARPP-32-negative cells. The enlargement of c-fos-positive cells is shown in the squares on the lower left. (C) Comparison of the density of c-fos-positive cells in the striatum between the control and cerebellar dystonia mice (*n*=12 in each group). (D) Comparisons of the ratio of DARPP-32-positive cells among the c-fos-positive cells (*n*=12 in each group). Box plots show the interquartile range, whiskers show the minimum and maximum values, and the median is marked with a line. n.s., not significant; **P<0.01; Mann–Whitney *U*-test.

**Fig. 3.**
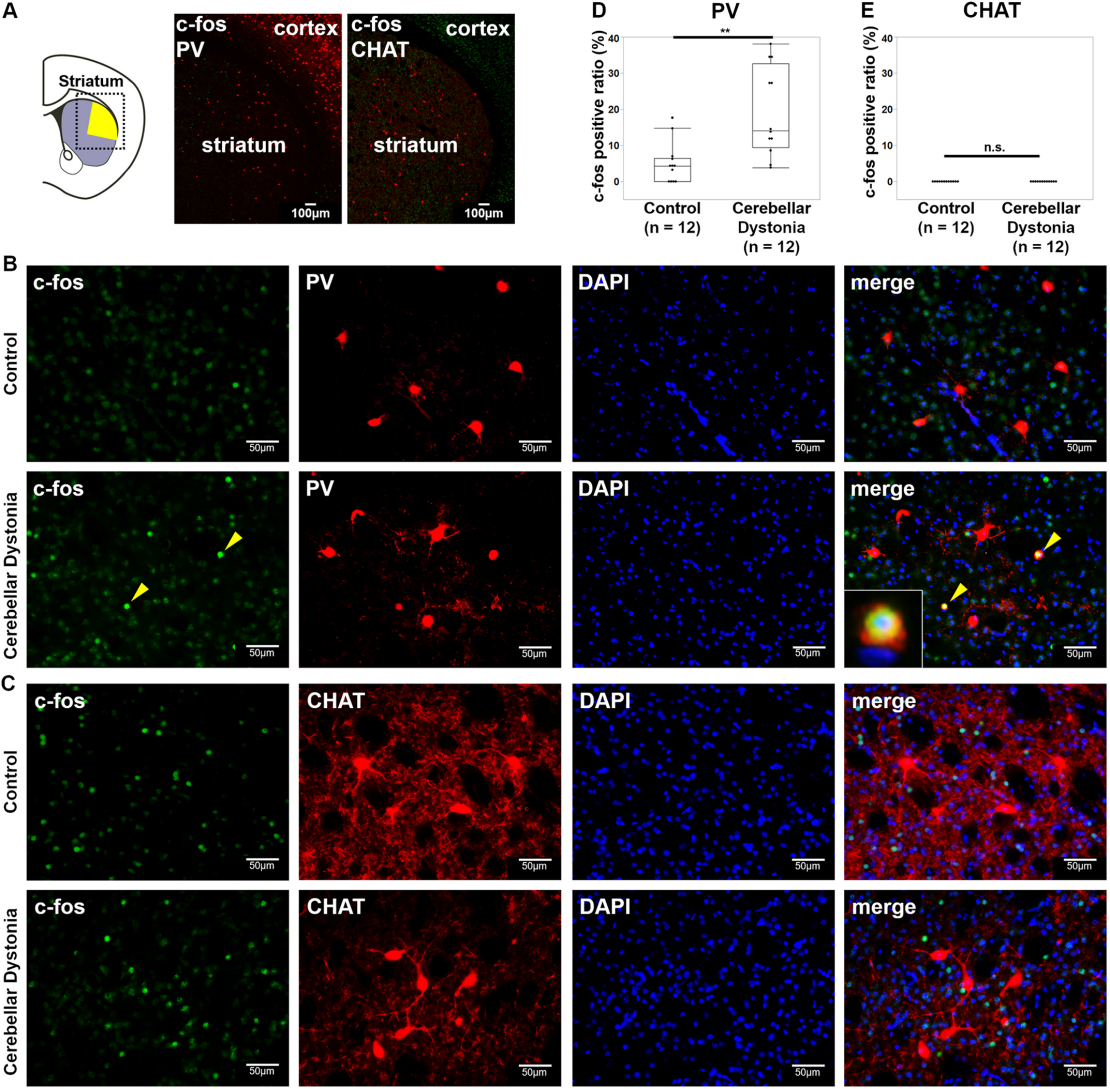
**c-fos-positive ratio of striatal PV and cholinergic interneurons.** (A) Schema of the striatum and immunostaining of c-fos (green) and parvalbumin (PV) (red) or choline acetyltransferase (CHAT) (red) in the square. The yellow area shows the dorsolateral striatum, which was determined according to a previous report (Morigaki et al., 2020). (B) Immunostaining of c-fos (green) and PV (red) in the striatum. The c-fos-positive PV interneurons are marked by yellow arrowheads. The enlargement of the c-fos-positive PV interneuron is shown in the square in the lower left. (C) Immunostaining of c-fos (green) and CHAT (red). (D) Comparisons of the c-fos-positive ratio in striatal PV and cholinergic interneurons between the control and cerebellar dystonia mice (*n*=12 in each group). Box plots show the interquartile range, whiskers show the minimum and maximum values excluding outliers, and the median is marked with a line. n.s., not significant; **P<0.01; Mann–Whitney *U*-test.

The activation of MSNs and interneurons in the striatum was examined. The schema of brain slices and immunostaining examples are shown in Fig. 2A,B. The size of the area in the dorsolateral striatum was the same between the control and cerebellar dystonia model mice (r=0.19, *P*=0.3408, Mann–Whitney *U*-test) (Fig. S1). No significant differences were found in the density of cells positive for c-fos (encoded by *Fos*) in the dorsolateral striatum (r=0.30, *P*=0.1410, Mann–Whitney *U*-test) (Fig. 2C). However, among the c-fos-positive cells in the dorsolateral striatum, the ratio of dopamine- and cAMP-regulated phosphoprotein of 32 kDa (DARPP-32, encoded by *Ppp1r1b*)-positive cells was higher in control mice than in cerebellar dystonia mice (r=0.64, *P*=0.0016, Mann–Whitney *U*-test) (Fig. 2D). This means that non-MSNs (i.e. some interneurons) were activated more in the cerebellar dystonia mice. The types of activated interneurons in the dorsolateral striatum were examined. Fig. 3A shows the schema and immunostaining of the striatal brain slices. Fig. 3B,C shows magnified images of immunostained striatal PV and cholinergic interneurons, respectively. No significant differences were observed in the density of PV interneurons between the two groups (r=0.03, *P*=0.8852, Mann–Whitney *U-*test) (Fig. S2). The cerebellar dystonia mice showed a higher ratio of c-fos-positive PV interneurons than that of the control mice (r=0.58, *P*=0.0046, Mann–Whitney *U-*test) (Fig. 3D). No c-fos-positive cholinergic interneurons were noted in either group (Fig. 3E). Based on these results, in mice with the cerebellar dystonia, PV interneurons were activated and MSNs were suppressed in the dorsolateral striatum.

### Activation of the entopeduncular nucleus, SNr and GPe in the cerebellar dystonia mice

**Fig. 4.**
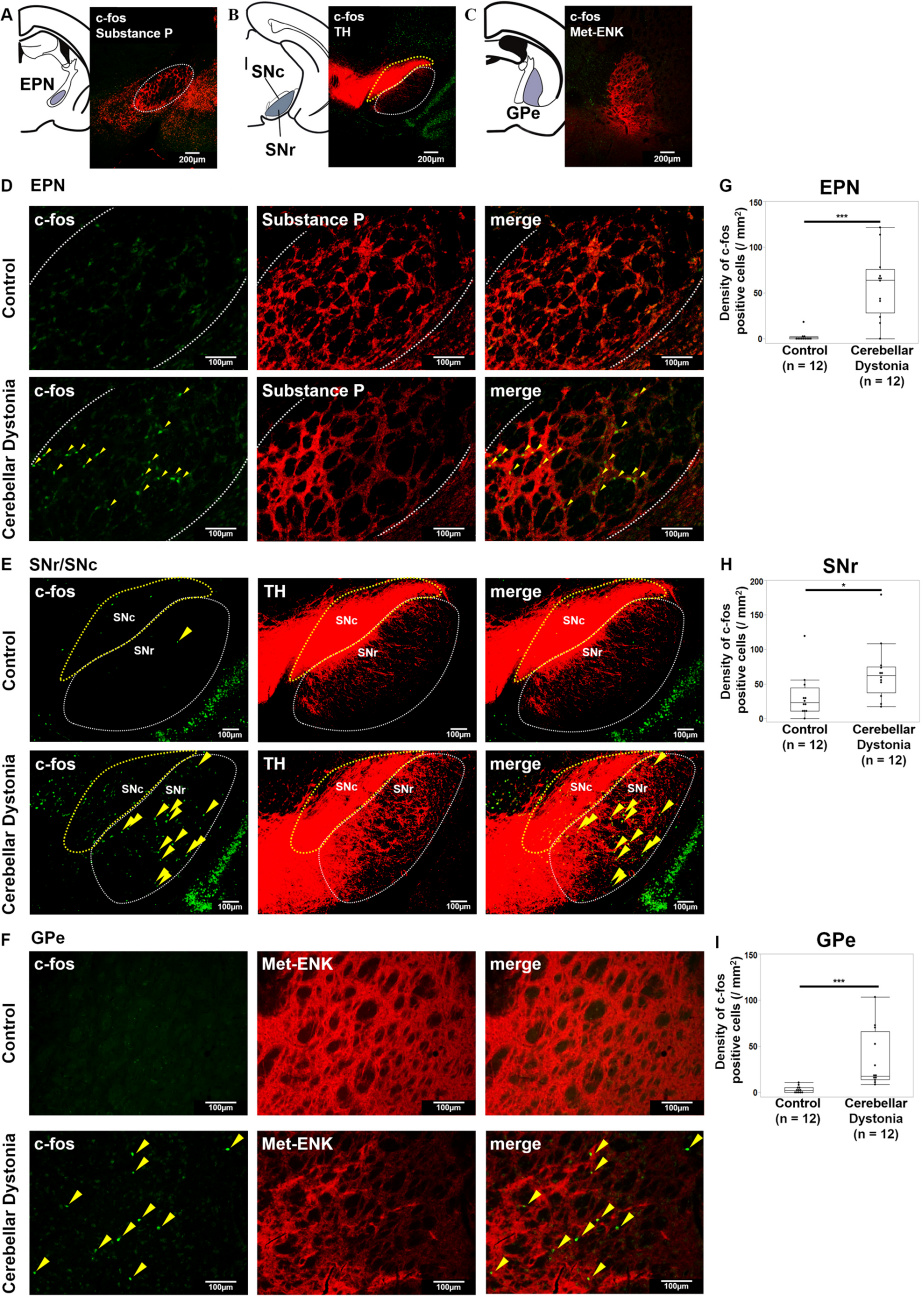
**Immunohistochemistry of EPN, SNr/SNc and GPe in the control and cerebellar dystonia mice.** (A-F) Schema of brain slices and immunostaining of the entopeduncular nucleus (EPN) using antibodies against c-fos (green) and substance P (encoded by *Tac1*) (red) (A,D), the substantia nigra pars reticulata (SNr) and substantia nigra pars compacta (SNc) using antibodies against c-fos (green) and tyrosine hydroxylase (TH) (B,E), and the globus pallidus externus (GPe) using c-fos (green) and methionine enkephalin (Met-ENK, encoded by *Penk*) (red) (C,F). The white dotted circles show the outlines of the EPN (D) and SNr (E), respectively, and the yellow ones show the outline of the SNc. The c-fos-positive cells are marked using yellow arrowheads. (E,F) Comparison of the number of c-fos-positive cells in the EPN (G), SNr (H) and GPe (I) between the control and cerebellar dystonia mice (*n*=12 in each group). Box plots show the interquartile range, whiskers show the minimum and maximum values excluding outliers, and the median is marked with a line. *P<0.05; ***P<0.001; Mann–Whitney *U*-test.

Next, we examined whether any change occurred in the anatomy downstream of the motor loop in cerebellar dystonia. Because striatal MSNs were suppressed, we investigated the anatomy downstream of the circuit: the entopeduncular nucleus (EPN), SNr, GPe and STN. No significant differences were found in the size of the examined areas of the EPN, SNr, GPe and STN between the groups (r=0.32, *P*=0.1124; r=0.26, *P*=0.2025; r=0.02, *P*=0.9310; and r=0.30; *P*=0.1406, respectively; Mann–Whitney *U-*test) (Fig. S3). The schema of brain slices and immunostaining examples are shown in Fig. 4A,D for the EPN, Fig. 4B,E for the SNr, Fig. 4C,F for the GPe, and Fig. S4A,B for the STN. In the EPN, SNr and GPe, more c-fos-positive cells were found in the cerebellar dystonia mice than in the control mice (r=0.77, *P*=0.0002; r=0.51, *P*=0.0120; and r=0.81, *P*≤0.0001, respectively; Mann–Whitney *U-*test) (Fig. 4G-I). The activation of cells in the EPN, SNr and GPe in the cerebellar dystonia mice could be due to the suppression of striatal dopamine D1- and dopamine D2-positive MSNs by overactivated PV interneurons. Immunostaining of the GPe in cerebellar dystonia with antibodies against c-fos and PV or c-fos and FoxP2 are shown in Fig. S5A,B, respectively. The ratio of PV-positive cells was higher than that of FoxP2-positive cells among c-fos-positive cells (r=0.83, *P*=0.0009, Mann–Whitney *U-*test) (Fig. S5C). No significant difference was found in c-fos density between cerebellar dystonia and control mice in the STN (r=0.03, *P*=0.8847, Mann–Whitney *U-*test) (Fig. S4C). These results suggest significant alterations in the activity of the basal ganglia in cerebellar dystonia.

### Combined administration of dopamine D1 receptor agonist and dopamine D2 receptor antagonist and selective ablation of striatal PV interneurons alleviate involuntary movements of the cerebellar dystonia mice

As the activation of PV interneurons could strongly suppress both striatal dopamine D1 receptor (DRD1)-type and dopamine D2 receptor (DRD2)-type neurons (Silberberg and Bolam, 2015), we hypothesized that a dopamine D1 receptor agonist (hereafter D1 agonist) and a dopamine D2 receptor antagonist (D2 antagonist) could effectively modulate involuntary movements in the cerebellar dystonia mice.

First, we conducted an experiment in which the D1 agonist and D2 antagonist (*n*=6), D1 agonist and saline (*n*=6), D2 antagonist and saline (*n*=6), and saline and saline (*n*=6) were administered to cerebellar dystonia mice, and compared dystonia rating scales between the ‘D1 agonist and D2 antagonist’ group and other groups. No significant differences were found after the correlation between them (‘D1 agonists and saline’, r=0.52, *P*=0.2199; ‘D2 antagonists and saline’, r=0.62, *P*=0.099; and ‘saline and saline’, r=0.68, *P*=0.0549; Mann–Whitney *U-*test) (Fig. S6). To compare the two groups ‘D1 agonist and D2 antagonist’ and ‘saline and saline’, the sample size was determined using Sample Size Explorers in JMP, with an α error of 0.05 and a power of 0.8, followed by performing another experiment between these two groups (*n*=8, respectively).

**Fig. 5.**
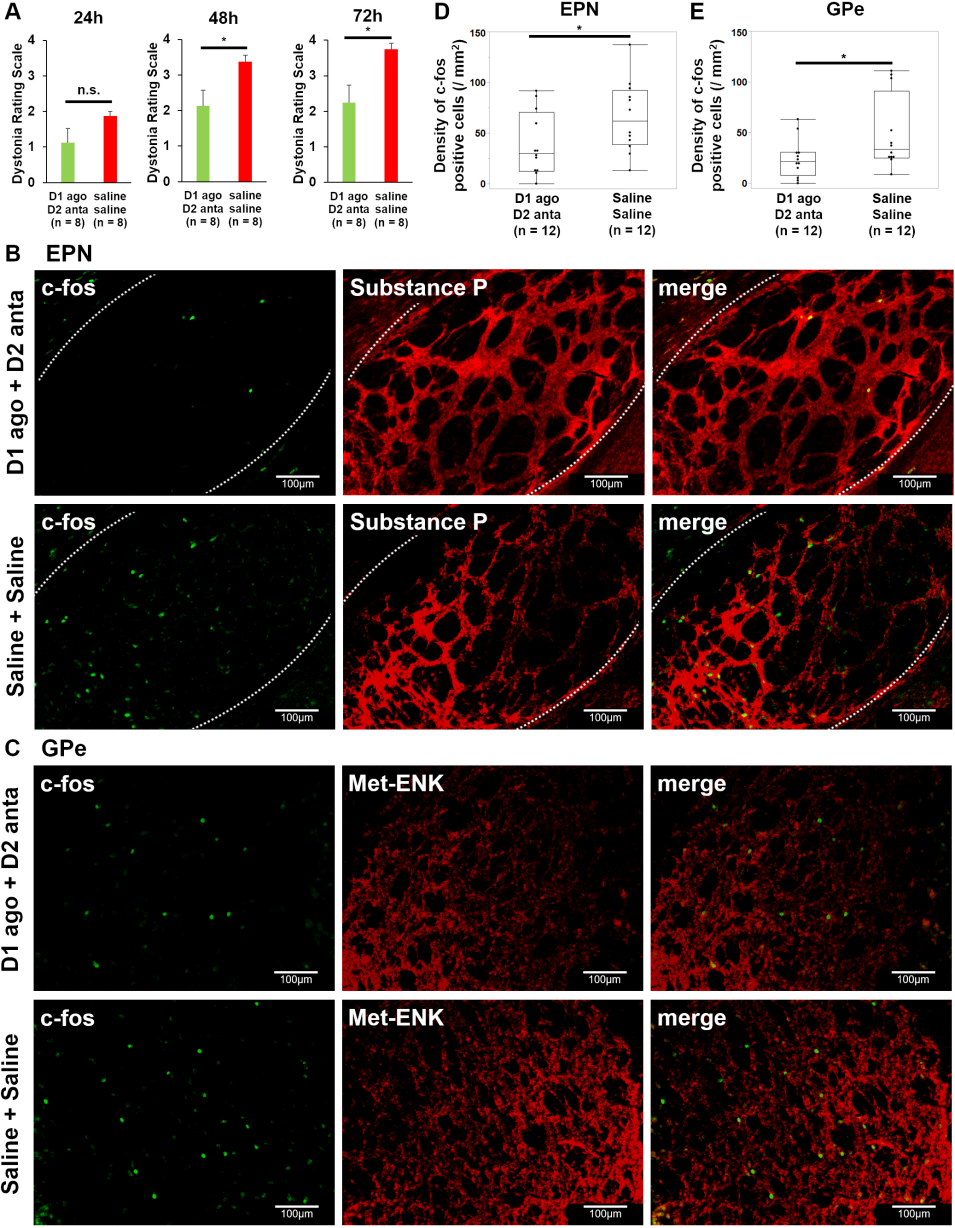
**Drugs administered for cerebellar dystonia mice.** (A) Changes in the dystonia rating scale of cerebellar dystonia mice that were administered dopamine D1 receptor agonists (‘D1 ago’) and D2 receptor antagonists (‘D2 anta’) or saline and saline (*n*=8 in each group). Bars represent the mean±s.e.m. (B,C) Immunostaining of the entopeduncular nucleus (EPN) using antibodies against c-fos (green) and substance P (red) (B), as well as of the globus pallidus externus (GPe) using antibodies against c-fos (green) and methionine enkephalin (Met-ENK) (red) (C) for the ‘D1 agonist and D2 antagonist’ and ‘saline and saline’ groups. (D,E) Comparisons of the number of c-fos-positive cells in the EPN (D) and GPe (E) between the ‘D1 agonist and D2 antagonist’ and ‘saline and saline’ groups (*n*=12 in each group). Box plots show the interquartile range, whiskers show the minimum and maximum values excluding outliers, and the median is marked with a line. n.s., not significant; *P<0.05; Mann–Whitney *U*-test.

At 72 h post operation, the dystonic symptoms of the ‘D1 agonist and D2 antagonist’ group were significantly reduced compared with those of the ‘saline and saline’ group (r=0.56, *P*=0.0261, Mann–Whitney *U-*test) (Fig. 5A; Movie 2).

The results of immunostaining of the EPN and GPe in cerebellar dystonia mice treated with D1 agonist and D2 antagonist are shown in Fig. 5B,C, respectively. The number of c-fos-positive cells was reduced more in the ‘D1 agonist and D2 antagonist’ group than in the ‘saline and saline’ group in both the EPN and GPe (r=0.41, *P*=0.0464 and r=0.41, *P*=0.0464, respectively; Mann–Whitney *U-*test) (Fig. 5D,E). Fig. S7A shows the immunostaining results of the c-fos-positive striatal PV interneurons. No significant difference was noted in the c-fos-positive ratio of striatal PV interneurons (r=0.26, *P*=0.2028, Mann–Whitney *U-*test) (Fig. S7B). Furthermore, selective ablation of striatal PV interneurons by a second immunotoxin was also effective in relieving involuntary movements of cerebellar dystonia 48 and 72 h after ouabain pump implantation (r=0.68, *P*=0.0067 and r=0.64, *P*=0.0101, respectively; Mann–Whitney *U-*test) (Fig. S8; Movie 3). These results suggest the involvement of the motor loop of basal ganglia in the pathogenesis of cerebellar dystonia and the potential for symptom improvement in cerebellar dystonia through the modulation of striatal neurons.

### Dopamine control of cerebellar dystonia mice

**Fig. 6.**
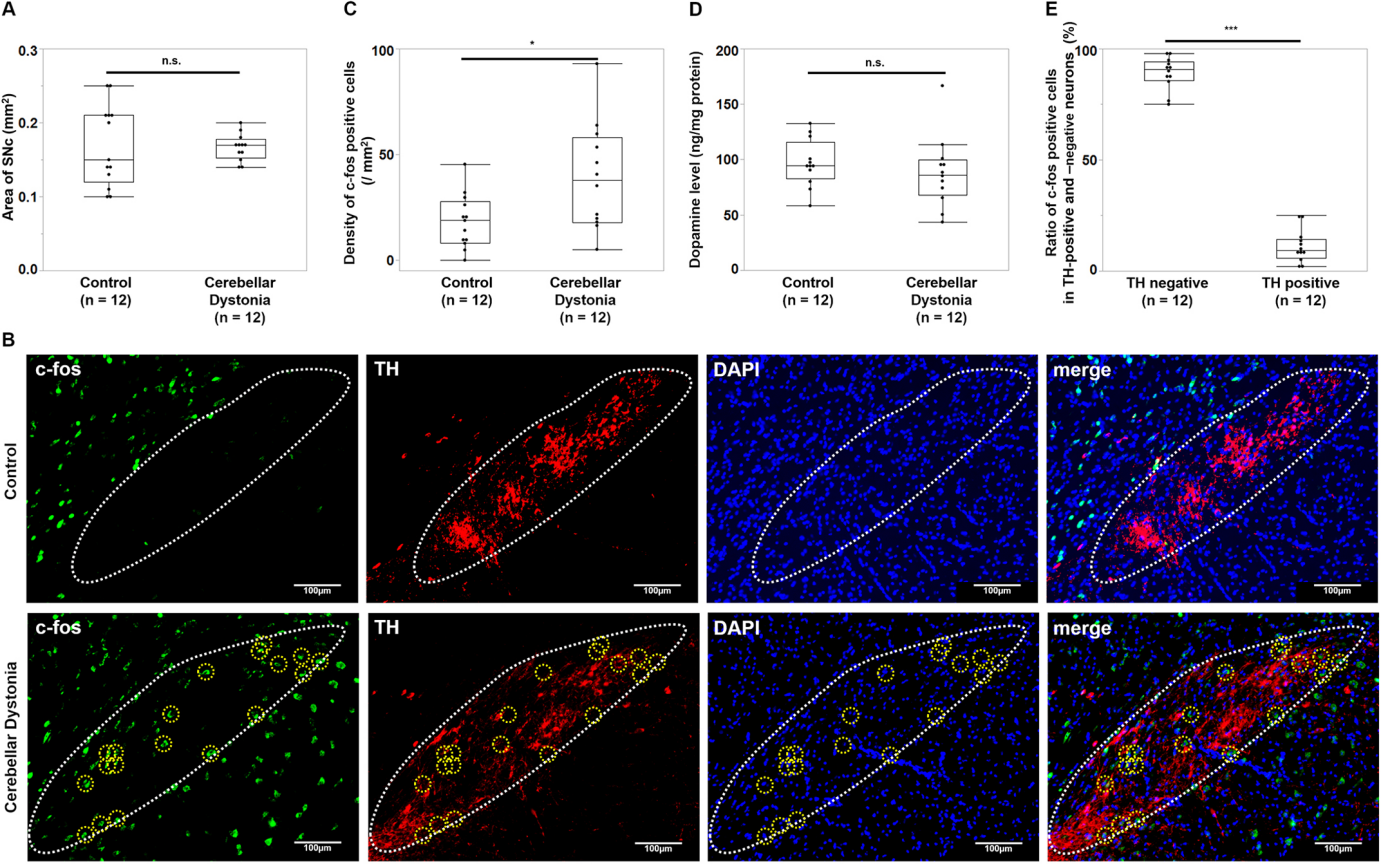
**Immunohistochemistry of SNc and dopamine levels in the striatum of the cerebellar dystonia mice.** (A) Comparisons of the area of substantia nigra pars compacta (SNc) (*n*=12 in each group). (B) Immunostaining of the SNc of the control and cerebellar dystonia mice. Yellow dotted circles show c-fos-positive and TH-negative cells and white dotted regions show SNc outlines. (C) Comparisons of the density of c-fos-positive cells in the SNc (*n*=12 in each group). (D) Comparisons of striatal dopamine levels between control and cerebellar dystonia mice (*n*=12 in each group). (E) Comparisons of the ratios of TH-negative and TH-positive cells among c-fos-positive cells in the SNc (*n*=12 in each group). Box plots show the interquartile range, whiskers show the minimum and maximum values excluding outliers, and the median is marked with a line. *P<0.05; ***P<0.001; Mann–Whitney *U*-test.

Dopamine control of cerebellar dystonia mice was evaluated by examining the activation of the substantia nigra pars compacta (SNc) and dopamine levels in the striatum. No significant differences were found in the size of the examined area in the SNc (r=0.02, *P*=0.9346, Mann–Whitney *U-*test) (Fig. 6A). Immunostaining examples of the SNc of the cerebellar dystonia and control mice are shown in Fig. 6B. The density of c-fos-positive cells in the SNc was higher in the cerebellar dystonia mice than in control mice (r=0.45, *P*=0.0276, Mann–Whitney *U-*test) (Fig. 6C); however, no significant differences were noted in striatal dopamine levels between groups (r=0.18, *P*=0.3708, Mann–Whitney *U*-test) (Fig. 6D). Among the c-fos-positive cells in the SNc, the ratio of tyrosine hydroxylase (TH)-negative cells was higher than that of TH-positive cells (r=0.84, *P*<0.0001, Mann–Whitney *U*-test) (Fig. 6E). Overall, non-dopaminergic neurons appeared to be more activated in the SNc of cerebellar dystonia, and no significant change was observed in dopamine levels within the striatum.

## DISCUSSION

### Mechanism underlying cerebellar dystonia

To the best of our knowledge, this study is the first to report the activation of striatal PV interneurons in cerebellar dystonia mice established through chronic infusion of ouabain, a Na^+^/K^+^ ATPase inhibitor, to the cerebellum, and the effectiveness of D1 agonist and D2 antagonist in modulating the involuntary movements induced by cerebellar dystonia. Furthermore, selective ablation of striatal PV interneurons alleviated involuntary movements. These results indicated that the activation of PV interneurons might not be a compensatory alteration.

**Fig. 7.**
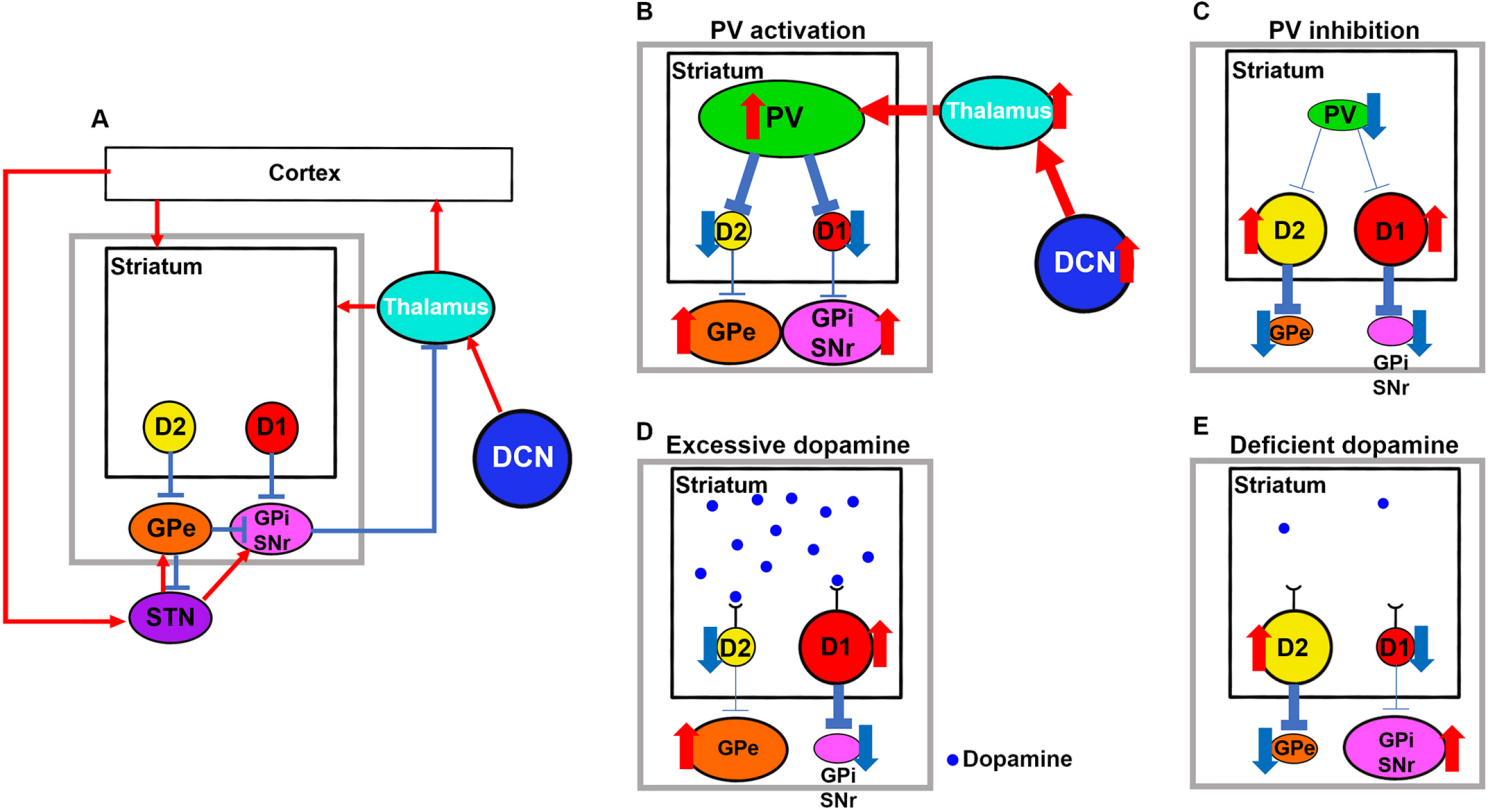
**Schema of our hypothesis of dystonia induced by disturbances in PV interneurons or dopamine levels.** (A) Normal situation of the cortico-basal ganglia–thalamo-cortical motor loop. The gray rectangle is expanded in B-E. (B-E) Effects of (B) parvalbumin (PV) interneuron activation (our new hypothesis), (C) PV interneuron inhibition, (D) excessive dopamine and (E) deficiency of dopamine. D1, dopamine D1 receptor; D2, dopamine D2 receptor; DCN, deep cerebellar nucleus; GPe, globus pallidus externus; GPi, globus pallidus internus; PV, parvalbumin interneuron; SNr, substantia nigra pars reticulata; STN, subthalamic nucleus.

In a previous report, the distributions of the injected drug involved the DCN (Calderon et al., 2011). Nakano et al. (2018) reported that the ventral anterior and ventral lateral complexes in the thalamus receive glutamatergic afferents from the DCN and send glutamatergic output to striatal PV interneurons. Consequently, depolarization of cells in the DCN by the Na^+^/K^+^ ATPase inhibitor could activate striatal PV interneurons. As striatal PV interneurons suppress MSNs (Silberberg and Bolam, 2015), neuronal hyperactivation in the EPN and GPe could have occurred in the cerebellar dystonia model. Fig. 7A shows the normal situation of the motor loop and Fig. 7B shows the hypothesized schema of cerebellar dystonia. The presence of multiple pathways from the cortex (hyperdirect pathway) (Nambu, 2004) and from the GPe to the STN might explain why there were no observable changes in c-fos expression in the STN. Approximately 20% of activated neurons in the GPe were FoxP2 positive in cerebellar dystonia (Fig. S5). Considering that FoxP2-positive cells occupy approximately 20% of total GPe neurons (Dong et al., 2021), there may be no cell type dependency regarding the neuronal activation between FoxP2- and PV-positive neurons.

Contrary to our hypothesis, Gittis et al. (2011) reported that selective inhibition of striatal PV interneurons resulted in dyskinesia. Involuntary movements are caused by an imbalance between the direct and indirect pathways (Damodaran et al., 2014). Furthermore, the synchronized firing of PV interneurons is crucial for maintaining the balance between the direct and indirect pathways (Damodaran et al., 2014). Accordingly, both overactivation and inhibition of striatal PV interneurons could cause involuntary movements. The inhibition of PV interneurons induces overactivation of both D1 neurons and D2 neurons, which results in the activation and inhibition of the direct and indirect pathways, respectively (Fig. 7C).

The discussion regarding the levels of dopamine in the striatum in dystonia is still ongoing. Both excessive and insufficient levels of dopamine can lead to dystonia/dyskinesia (Breakefield et al., 2008), although there are also findings supporting normal levels in dystonia (Eidelberg et al., 1993; Kawarai et al., 2017). In cases of excessive dopamine levels, such as dopamine-induced dyskinesia in Parkinson's and Huntington's diseases, D1 and D2 neurons are activated and inhibited, respectively, by overstimulation of D1 and D2 receptors (Morigaki and Goto, 2017; Morigaki et al., 2017), which activates both the direct and indirect pathways (Fig. 7D). Contrastingly, deficient dopamine levels cause dopamine-responsive dystonia (DYT5-GCH1) and off-dystonia in patients with Parkinson's disease (Breakefield et al., 2008). In this condition, D1 cells are inhibited and D2 cells are activated due to reduced stimulation of D1 and D2 receptors, which inhibits both the direct and indirect pathways (Fig. 7E).

Disturbances in the activity of striatal PV interneurons are as important as the amount of available dopamine for generating the imbalance between the two pathways. Considering the activation of GPe, EPN and SNr in both the direct and indirect pathways, our findings suggest that the imbalance between these pathways, primarily driven by the activation of PV interneurons rather than changes in dopamine levels, is responsible for inducing involuntary movements in cerebellar dystonia (Fig. 7).

Hisatsune et al. (2013) suggested that cerebellar dystonia was induced by tonic Purkinje cell firing independent of the basal ganglia as there was no altered striatal c-fos expression in the mouse model of cerebellar dystonia. However, they did not examine the types of activated cells. In our results, no significant difference was found in the number of c-fos-positive cells; however, the types of activated cells were different. Specifically, PV interneurons were activated in cerebellar dystonia mice, whereas MSNs were activated in the control mice. This might explain the discrepancy between our result and that of Hisatsune et al. (2013).

### Difference between cerebellar and non-cerebellar dystonia

Activation of cholinergic interneurons could occur in dystonia; accordingly, anti-cholinergic drugs are effective therapeutic agents for dystonia (Eskow Jaunarajs et al., 2015; Jankovic, 2013). However, we did not observe activation of cholinergic interneurons; rather, we observed unexpected activation of PV interneurons. This suggests that the activation of PV interneurons, instead of cholinergic interneurons, could be important in cerebellar dystonia. Accordingly, anti-cholinergic drugs might not have a direct effect on cerebellar dystonia. There are differences in the correlograms of PV and cholinergic interneurons to MSNs (Adler et al., 2013). In addition, Gritton et al. (2019) suggested that ill-timed PV activation might cause hyperkinetic involuntary movements as PV interneurons could trigger new movements and modulate the trajectory of ongoing movement. Furthermore, they found that cholinergic interneurons could control movements by triggering the end of a movement bout.

Although pallidal DBS is a major treatment strategy for dystonia, the effectiveness of GPi-DBS is dependent on the type of dystonia; specifically, tonic dystonia is frequently refractory, whereas phasic dystonia shows good improvement. Yokochi et al. (2018) hypothesized that the tonic and phasic components originate from cerebellar dysfunction and the basal ganglia, respectively, which could be attributed to differences in the types of interneurons that are overactivated. Accordingly, we adopted a different approach and investigated cerebellar dystonia based on its differential pathogenesis; tonic and phasic dystonia might be caused by a disturbance in PV and cholinergic interneurons, respectively.

### Drugs for treating cerebellar dystonia

We observed the activation of the striatal PV interneurons, EPN and GPe in the mouse model of cerebellar dystonia. We chose D1 agonists and D2 antagonists as drugs for modulating cerebellar dystonia in our study. Compared with previous studies (Gerfen et al., 2002; Paul et al., 1992), the present study used a sufficiently large dose of these drugs. They relieved involuntary movements induced by cerebellar dystonia. Furthermore, selective ablation of PV interneurons also alleviated the involuntary movements. These results imply the crucial involvement of striatal neurons in the pathogenesis of cerebellar dystonia.

Calderon et al. (2011) reported that the ablation of the thalamic centrolateral nucleus could ameliorate dystonia worsening using the present mouse model. Although the thalamic ventral anterior–ventral lateral and intralaminar nuclei are the only nuclei with an established relationship with PV interneurons (Sciamanna et al., 2015; Nakano et al., 2018; Assous and Tepper, 2019; Martel and Galvan, 2022), the centrolateral nucleus might also have excitatory neural connections with striatal PV interneurons. As the thalamo-striatal projections are heterogenous and the striatum receives glutamatergic inputs from numerous thalamic nuclei (Hintzen et al., 2018; Mengual et al., 1999; Nakano et al., 2018), the relationship between the thalamic nucleus and striatal PV interneurons should be elucidated further. Suppressing PV interneurons by modulating the thalamic nucleus or DCNs might be an effective treatment strategy.

Certainly, in addition to the DCN-thalamus-striatum pathway we have discussed, there are other connections of the DCN with the striatum to consider. The disynaptic pathways via the ventral tegmental area and SNc to the striatum are important as the DCN controls dopamine release through these pathways (Yoshida et al., 2022). In our findings, non-dopaminergic neurons appeared activated in the SNc; however, no significant changes were observed in the overall dopamine levels within the striatum. Further investigation using more detailed dopamine measurement techniques to explore localized and temporal alterations in dopamine levels will be necessary in future studies.

### Characteristics of cerebellar dystonia

As activated PV interneurons strongly suppress striatal D1 and D2 cells (Silberberg and Bolam, 2015), with activation of both the EPN and GPe, the direct pathway should be suppressed and the indirect pathway should be activated in cerebellar dystonia (Fig. 7B). The direct and indirect pathways might determine the amount (quantity) and accuracy (quality) of movements, respectively (Amemori et al., 2011; Morigaki et al., 2021). Therefore, the involuntary movements in cerebellar dystonia could be described as hypokinesis with concomitant unwanted movements. In our study, the total moving distance and average velocities reflected the degree of movements, and dystonia rating scales reflected the accuracy of the movements.

### Dopamine control in cerebellar dystonia

In our study, TH-negative cells were activated in the SNc. Considering that the SNc is composed of dopamine neurons and GABAergic interneurons (Oorschot, 2010), GABAergic interneurons might be activated dominantly. Oh et al. (2017) reported that PV neurons in the GPe suppress dopamine cells in the SNc. Neychev et al. (2008) reported that dopamine levels were reduced in tottering mutant mice, which is established as a genetic cerebellar dystonia model. PV neurons in the GPe were activated in cerebellar dystonia mice in our study, and they might consequently suppress dopamine neurons in the SNc. Furthermore, the DCN projects directly to the SNc and controls the release of dopamine (Yoshida et al., 2022). Dopamine levels in the striatum did not change in our study. We measured dopamine levels in the whole striatum because the dorsolateral striatum was relatively small to separate from the whole striatum. Furthermore, we did not assess real-time dopamine change. Therefore, the precise dopamine release in cerebellar dystonia remains to be elucidated. A technique that can measure real-time and local dopamine levels, such as cyclic voltammetry, should be considered to elucidate this problem.

### Conclusions

Cerebellar dystonia could be induced through changes in the basal ganglia circuit. Striatal PV, rather than cholinergic, interneurons were activated in cerebellar dystonia mice. Modulating the direct and indirect pathways could be effective in alleviating their involuntary movements.

## MATERIALS AND METHODS

### Mouse model of cerebellar dystonia

All animal experiments complied with the ARRIVE guidelines, were approved by the Ethics Committee of Tokushima University (T2020-35) and were performed according to Directive 2010/63/EU.

We established a mouse model of cerebellar dystonia as previously described (Calderon et al., 2011). Ten-week-old male C57BL/6J mice (RRID: IMSR_JCL:MIN-0003, CLEA Japan, Tokyo, Japan) were randomly distributed in each group before the operation. The mice were individually housed (one per cage) in a plastic cage with a temperature- and humidity-controlled environment and a 12 h/12 h light/dark cycle. They had *ad libitum* access to food and water. Briefly, the mice were anesthetized with isoflurane (099-06571, FUJIFILM, Tokyo, Japan) and their heads were fixed to a stereotaxic instrument (SR-5M, Narishige, Tokyo, Japan).

After the skin incision, the skulls were perforated. In the dystonia model (*n*=8), ouabain (630-60-4, Tocris Bioscience, Bristol, UK) was diluted in saline and administered at 18 ng h^−1^ using an osmotic pump (1002, Alzet, Cupertino, CA, USA) and cannulae (3280PM/SPC, P1 Technologies, Roanoke, VA, USA). Using a micromanipulator (SMM-100, Narishige), the cannula tips were adjusted in the cerebellum [along the anterior-posterior (AP) axis: −6.9 mm from the bregma; along the medial-lateral (ML) axis: ±0 mm; along the ventral-dorsal (VD) axis: 3 mm] and fixed with dental cement (10013653 and 10013654, GC Corporation, Tokyo, Japan). The osmotic pumps were implanted under the skin on their back. For control mice (*n*=8), we used the same volume of saline in place of ouabain. The behavior of the mice was analyzed at 24 h intervals postoperatively. Mice were sacrificed at 72 h post operation. Hematoxylin and Eosin staining was performed to confirm the location of the tip of the pump, and 0.5% Evans Blue was used to confirm the distribution of the drugs. Fig. S9A shows the study protocol.

### Behavior evaluation and analysis

The behavior of the mice was analyzed using the dystonia rating scale and open-field test. Involuntary movement was evaluated using the dystonia rating scale (0, none; 1, inconsequential; 2, mild; 3, moderate; and 4, severe impairment) (Jinnah and Hess, 2015) by two observers masked to the group allocation and the mean of their scores was used as the final score.

In the open-field tests, mice were released in a circle with a diameter of 50 cm. After 15 min of free running, locomotion was recorded for 10 min using a video camera. The total moving distance was calculated. Furthermore, velocities at 0.03-s intervals were calculated, and the average was calculated. Video analysis was performed by Dartfish (RRID: SCR_023501, Dartfish Japan, Tokyo, Japan). At 72 h post operation, each parameter was compared between the cerebellar dystonia (*n*=8) and control (*n*=8) mice.

### Tissue preparation

Immediately after behavioral evaluation, mice were deeply anesthetized using an intraperitoneal injection of pentobarbital (SOM04-YO1706, Kyoritsu Seiyaku, Tokyo, Japan). They were perfused with saline, followed by 4% paraformaldehyde in phosphate buffer solution (163-20145, FUJIFILM). Subsequently, their brains were extracted and stored in 4% paraformaldehyde in phosphate buffer solution for 24 h for post fixation and then transferred into 10, 20 and 30% sucrose gradients in phosphate-buffered saline (PBS) at 24-h intervals at 4°C. We prepared 20-μm sections using a microtome (CM 1860, Leica, Nussloch, Germany) and stored them in PBS containing 0.05% sodium azide at 4°C until use.

The stained sections were chosen as follows: AP: +0.86 mm for the striatum; −1.34 mm from the bregma for the EPN, which is homologous to the GPi; −3.16 mm for the SNr and SNc; −0.70 mm for the GPe; and −1.70 mm for the STN. One slice from one mouse was used for each group.

### Immunohistochemistry assay

We immunostained free-floating brain sections using the tyramide signal amplification (TSA) method, as previously described (Morigaki and Goto, 2017). After the endogenous peroxidase activity of the sections was blocked using a 3% hydrogen peroxide solution, the sections were incubated in PBS containing 3% bovine serum albumin (PBS-BSA) for 1 h. Subsequently, the sections were incubated overnight with primary antibodies diluted in PBS-BSA. Table S1 shows details regarding the primary antibodies. After incubation in Histofine Simple Stain Kit (414341F, Nichirei Biosciences, Tokyo, Japan) for 45 min, the sections were treated for 15 min using the TSA system with fluorescein (NEL741001KT, Akoya Biosciences, Marlborough, MA, USA). For double staining, the sections were incubated in 0.1 M glycine-hydrogen chloride (HCl) (pH 2.2) before being processed in the same way as in the first staining. Cyanine 3 (NEL744001KT, Akoya Biosciences) was used for detection. A negative control is shown in Fig. S10. Anti-TH was used as a marker for the SNr and SNc, and anti-DARPP-32 was used as a marker for MSNs. The location of the STN was confirmed using anti-NeuN. We used anti-substance P and anti-methionine enkephalin as markers of direct and indirect pathways, respectively (Kalanithi et al., 2005).

Slices were observed using the Keyence BZ-X710 microscope (RRID: SCR_017202, Keyence, Osaka, Japan), with the number of positive cells being counted using BZ-X Analyzer Software (RRID: SCR_017375, Keyence). First, we counted the number of c-fos-positive cells in the dorsolateral striatum, and the ratio of DARPP-32 positive cells among them was calculated in the cerebellar dystonia (*n*=12) and control (*n*=12) mice. Segmentation of the dorsolateral striatum was performed according to Morigaki et al. (2020). Next, we counted the number of striatal PV and cholinergic interneurons, followed by the number of c-fos-positive interneurons among them in both the cerebellar dystonia (*n*=12) and control (*n*=12) mice. We calculated the c-fos-positive ratio of PV and cholinergic interneurons and compared the ratio between the two groups. Next, we counted the number of c-fos-positive cells in the EPN, SNr, GPe and STN of the cerebellar dystonia (*n*=12) and control (*n*=12) mice. Because the GPe contains two types of neurons, PV-expressing prototypic neurons and FoxP2-expressing arkypallidal neurons (Fujiyama et al., 2016), PV- and FoxP2-positive ratios among the c-fos-positive neurons in the GPe were calculated in the cerebellar dystonia mice (*n*=8, respectively). Regarding the SNc, the ratios of TH-positive and TH-negative cells among c-fos-positive cells were calculated and compared (*n*=12, respectively).

### Drug treatments

Next, we examined whether dopamine D1 agonists and/or D2 antagonists could modulate involuntary movements. We used SKF 81297 (1447, Tocris Bioscience) as a D1 agonist and raclopride (1810, Tocris Bioscience) as a D2 antagonist. The drugs were peritoneally administered at a concentration of 3 mg kg^−1^ day^−1^ using osmotic pumps (1003D, Alzet). The dose of the drugs was determined according to previous reports (Gerfen et al., 2002; Paul et al., 1992). As a preliminary experiment, the drug combinations were (1) D1 agonist and D2 antagonist, (2) D1 agonist and saline, (3) D2 antagonist and saline, and (4) saline and saline (*n*=6 each), followed by the establishment of the mouse model of cerebellar dystonia. The amount of DMSO required to dissolve the drugs was equal to the saline preparations for standardization. After the preliminary experiment, we conducted the main experiment in the ‘D1 agonist and D2 antagonist’ and ‘saline and saline’ groups in sufficient numbers (*n*=8, respectively). Mice were evaluated using dystonia rating scales at 72 h post operation. Immunostaining was performed and evaluated for tissues obtained from mice in the ‘D1 agonist and D2 antagonist’ and ‘saline and saline’ groups (*n*=12, respectively). Fig. S9B shows the study protocol.

### Selective ablation of dorsolateral striatal PV interneurons

Next, we examined the selective ablation of dorsolateral striatal PV interneurons using a second immunotoxin to relieve their involuntary movements. We used Streptavidin-ZAP (KIT-27-Arb100, Advanced Targeting Systems, Carlsbad, CA, USA), which is a conjugate between saporin and streptavidin. Streptavidin can bind biotinylated antibodies, and when the complex binds the target cells, conjugated saporin induces cell death (Ancheta et al., 2023). Streptavidin-ZAP was mixed with an anti-PV antibody (ab181086, Abcam, Cambridge, UK), which was biotinylated with a biotinylation kit (LK03, DOJINDO) in equimolar concentrations. They were diluted with PBS by 1:100, and 3 µl was injected into six sites in the striatum of mice (AP: +1.2 mm from the bregma, ML: ±1.8 mm, VD: 3.0 mm; AP: +0.7 mm from the bregma, ML: ±2.0 mm, VD: 3.4 mm; and AP: −0.1 mm from the bregma, ML: ±2.7 mm, VD: 3.0 mm) within 5 min. As a control, Control Molecules in the kit were used instead of Streptavidin-ZAP (*n*=8, respectively). After 72 h from the toxin injection, the ouabain pump and cannula were implanted to induce cerebellar dystonia, and they were evaluated using dystonia rating scales at 144 h post operation (i.e. 72 h after pump implantation) (*n*=8, respectively). Fig. S9C shows the study protocol.

### Measurement of striatal dopamine levels

The striatum of the cerebellar dystonia and control mice (*n*=12, respectively) was dissected, weighed and homogenized in lysis buffer containing 0.01 N HCl, 1 mM EDTA, and 4 mM sodium metabisulfite. After incubation on ice for 1 h, the samples were centrifuged at 13,000 ***g*** for 15 min at 4°C. The supernatants were collected, followed by measurement of dopamine levels using a Dopamine ELISA kit (BA-E-5300R, Immusmol SAS, Bordeaux, France) according to the manufacturer's instruction.

### Statistical analysis

In a preliminary experiment, we immunostained six GPe specimens (three control and cerebellar dystonia mice, respectively) and determined that a sample size of 24 was required for immunohistochemistry using Sample Size Explorers in JMP (RRID: SCR_014242, SAS Institute, NC, USA), with an α error of 0.05 and a power of 0.8.

We assessed our data for normality using the Shapiro–Wilk test before statistical analysis. Because we could not verify normality, we used the non-parametric Mann–Whitney *U*-test to analyze all our data. We included all data, and no data points were excluded as outliers. All statistical analyses were performed using JMP 14.0.0. The Mann–Whitney *U-*test was used for between-group comparisons. Dunnett's correction was used when multiple comparisons were performed, with the *P*-values being reported after adjustment. Statistical significance was set at *P*<0.05.

## Supplementary Material

10.1242/dmm.050338_sup1 Supplementary information
