# Supplementary material for: Striatal parvalbumin interneurons are activated in a mouse model of cerebellar dystonia
Source: Dis Model Mech. 2024 May 14;17(5):dmm050338. doi: 10.1242/dmm.050338 (PMC11128288; doi:10.1242/dmm.050338)
Supplement: Supplementary information [file dmm-17-050338-s1.pdf]

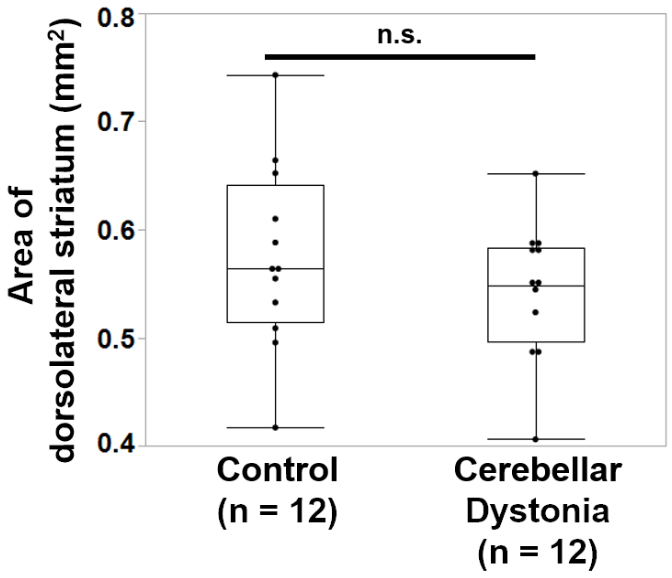

Fig. S1. Comparisons of the area of dorsolateral striatum.
Comparisons of the area of dorsolateral striatum between the control and cerebellar dystonia mice (n = 12 in each group). not significant; Mann–Whitney *U* test

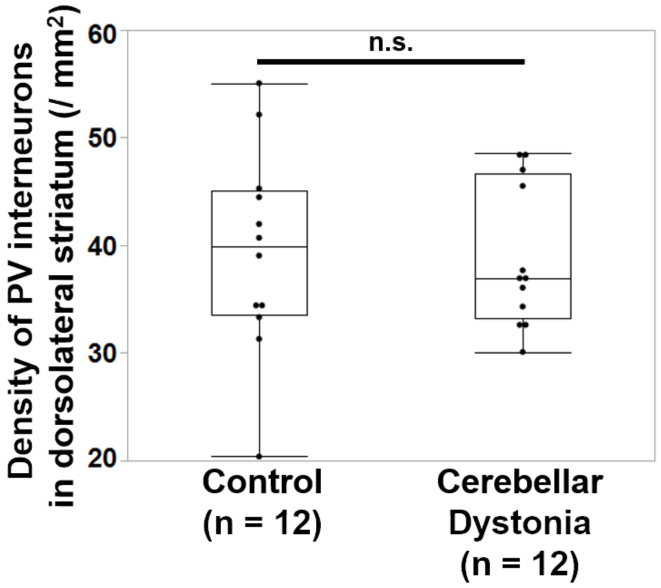

Fig. S2. Comparisons of the density of parvalbumin interneurons in dorsolateral striatum
Comparisons of the density of parvalbumin interneurons in the dorsolateral striatum between the control and cerebellar dystonia mice (n = 12 in each group). not significant; Mann–Whitney *U* test

Disease Models & Mechanisms • Supplementary information

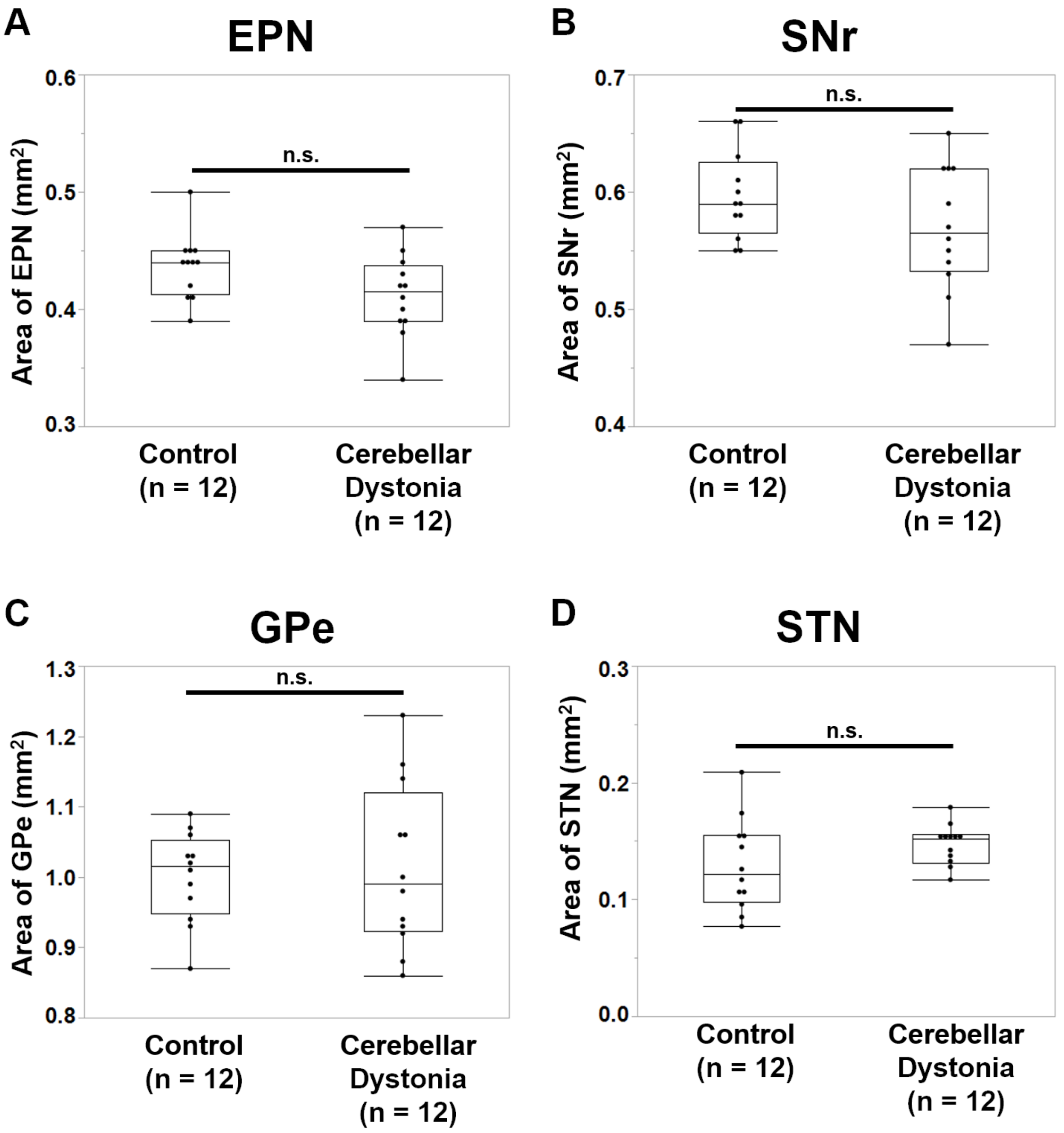

Fig. S3. Comparisons of the area of entopeduncular nucleus, substantia nigra pars reticulata, globus pallidus externus, and subthalamic nucleus. Comparisons of the area of the entopeduncular nucleus (A), substantia nigra pars reticulata (B), globus pallidus externus (C), and subthalamic nucleus (D) between the control and cerebellar dystonia mice (n = 12 in each group). not significant; Mann–Whitney *U* test

Abbreviations: EPN, entopeduncular nucleus; SNr, substantia nigra pars reticulata; GPe, globus pallidus externus; STN, subthalamic nucleus

Disease Models & Mechanisms • Supplementary information

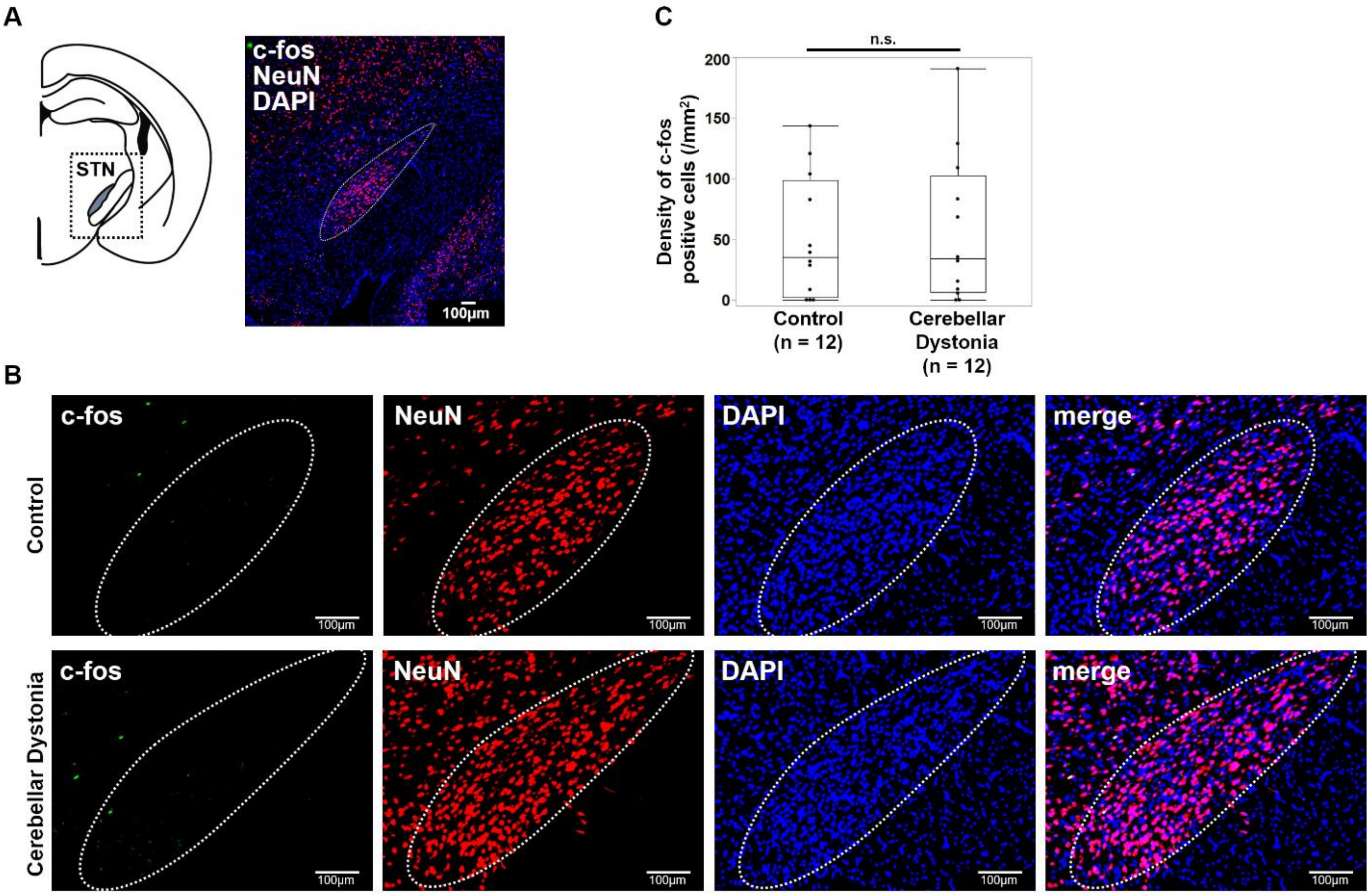

Fig. S4. Immunohistochemistry of the STN in the control and cerebellar dystonia mice

A, B: Schema of brain slices and immunostaining of the subthalamic nucleus (STN) using c-fos (green), NeuN (red), and DAPI (blue). The white dotted circles show the outline of the STN. C: Comparisons of the density of c-fos-positive cells in the STN (n = 12 in each group). n.s., not significant; Mann–Whitney *U* test

Abbreviations: STN, subthalamic nucleus; DAPI, 4′,6-diamidino-2-phenylindole

Disease Models & Mechanisms • Supplementary information

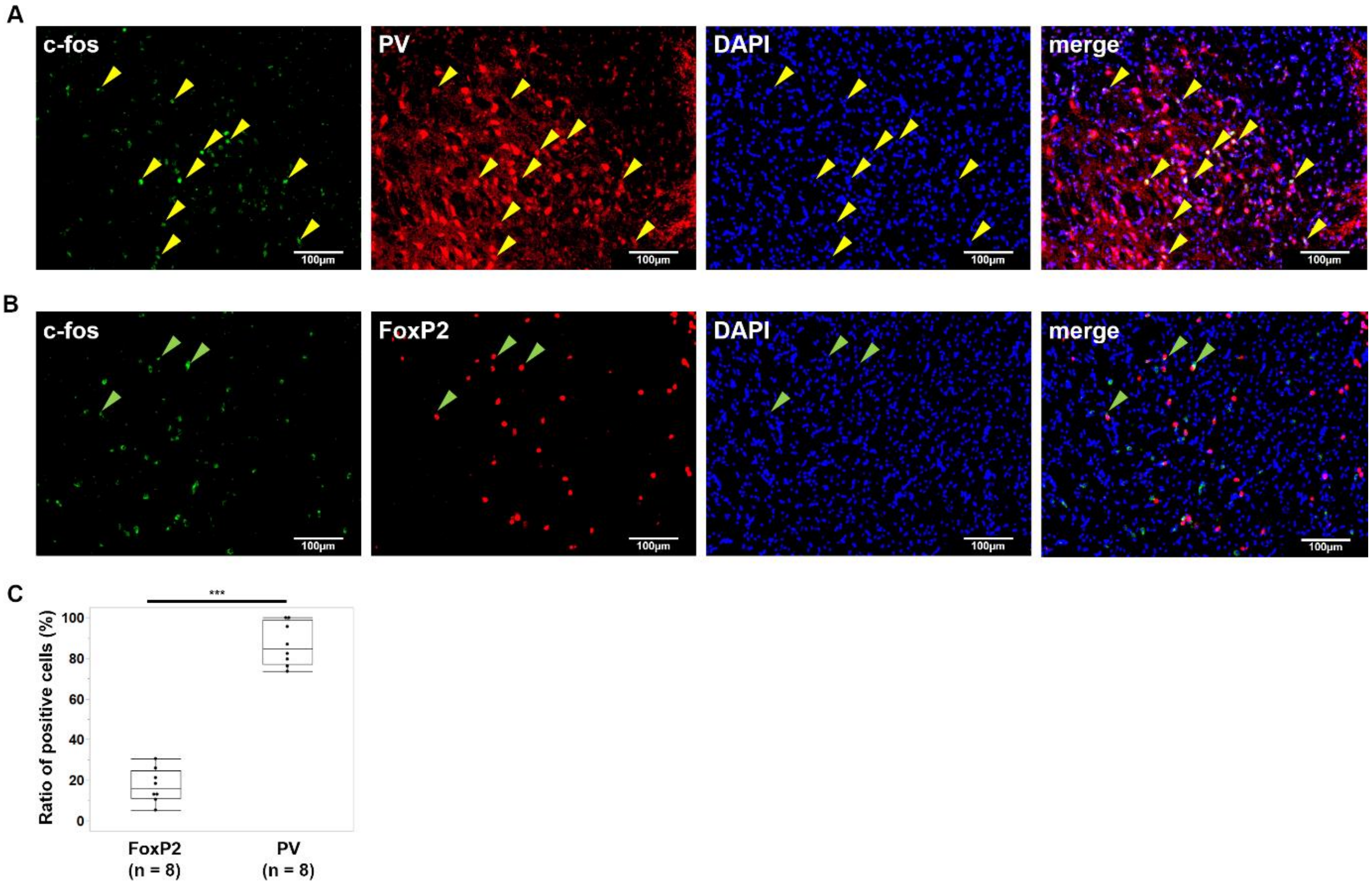

Fig. S5. Comparisons between the positive ratio of FoxP2- and PV-positive cells among c-fos-positive cells in the globus pallidus externus of the cerebellar dystonia mice.

A: Immunostaining of the globus pallidus externus (GPe) of the cerebellar dystonia mice using c-fos (green) and parvalbumin (PV) (red). B: Immunostaining of the GPe of the cerebellar dystonia mice using c-fos (green) and FoxP2 (red). C: Comparisons between the positive ratio of FoxP2- and PV-positive cells among c-fos-positive cells in the GPe of the cerebellar dystonia mice (n = 8 in each group). ***$p < 0.001$; Mann–Whitney *U* test

Abbreviations: PV, parvalbumin; DAPI, 4′,6-diamidino-2-phenylindole

Disease Models & Mechanisms • Supplementary information

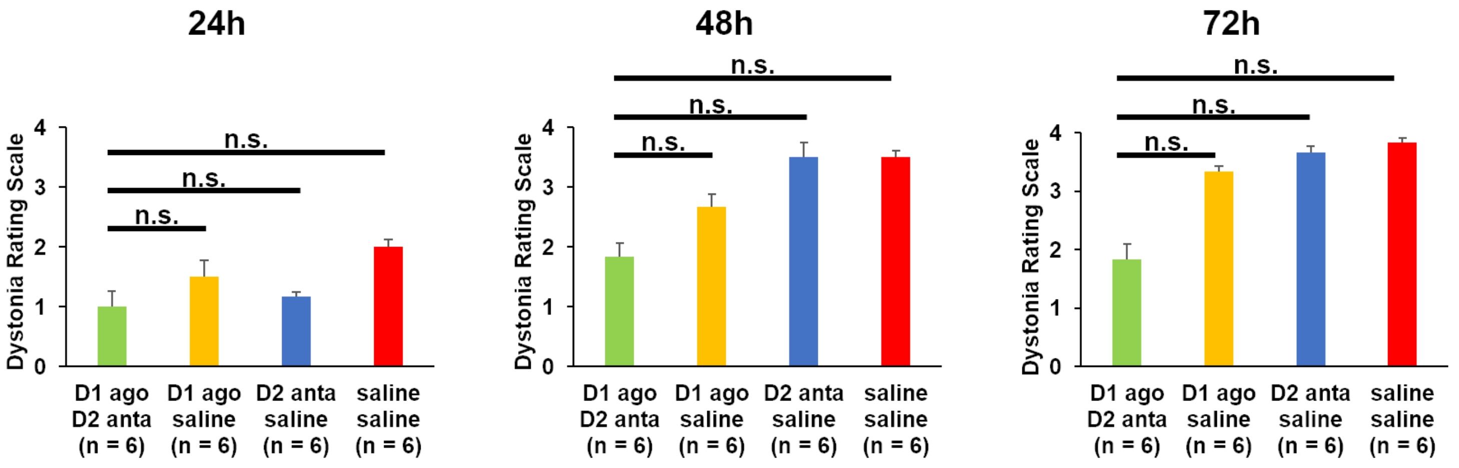

Fig. S6. The result of the preliminary experiment of drug administration.

Comparisons of dystonia rating scale scores at 24, 48, and 72 postoperative hours among the following four groups of drug administration: “D1 agonist and D2 antagonist,” “D1 agonist and saline,” “D2 antagonist and saline,” and “saline and saline” (n = 6 in each group). not significant; Mann–Whitney *U* test

Abbreviations: D1 ago, dopamine D1 agonist; D2 anta, dopamine D2 antagonist

Disease Models & Mechanisms • Supplementary information

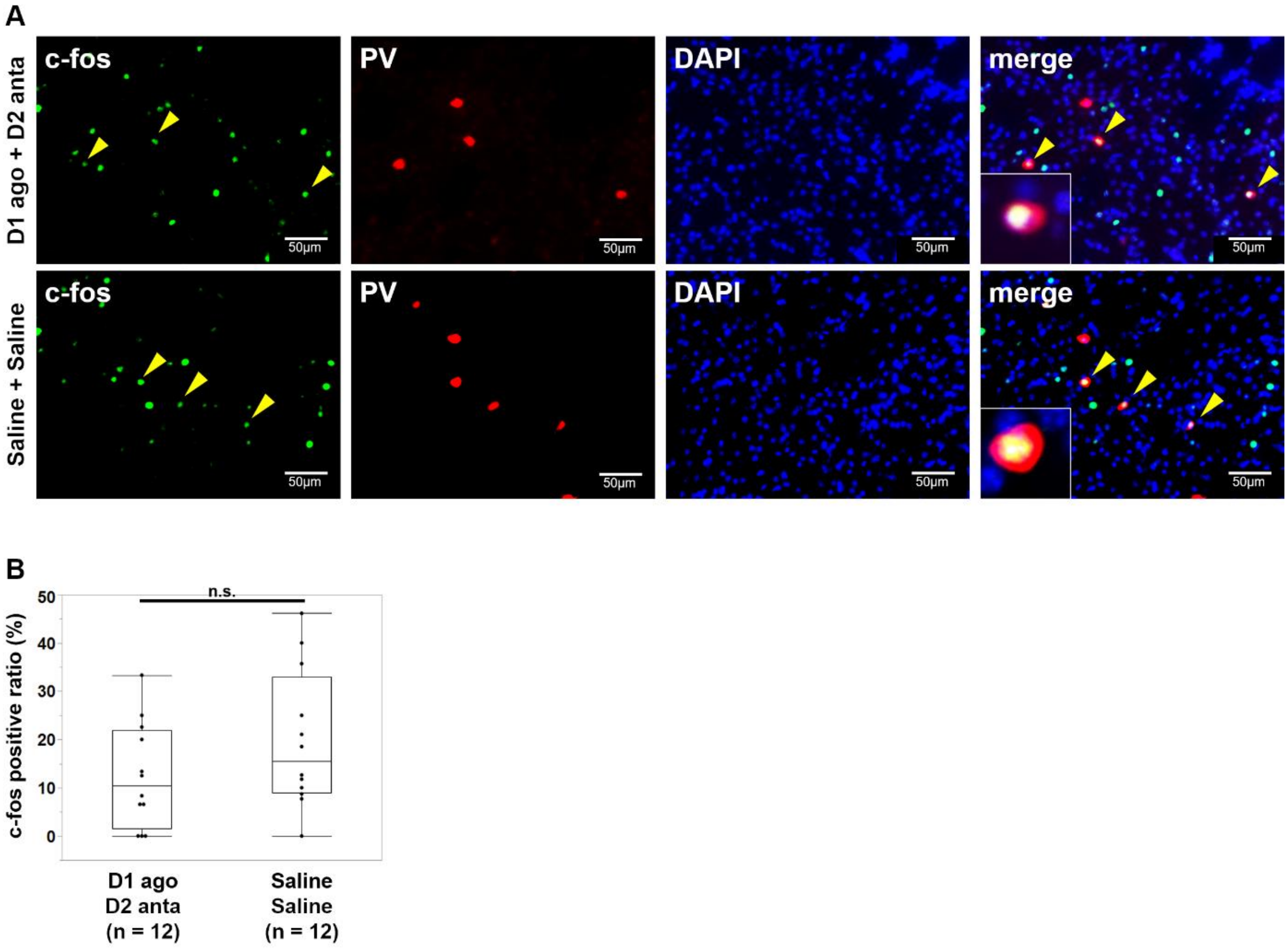

Fig. S7. The c-fos-positive ratio of striatal PV interneurons in the drug-treated mouse model of cerebellar dystonia.

A: Immunohistochemistry of the striatum using c-fos (green) and parvalbumin (PV) (red) of cerebellar dystonia mice administered “Dopamine D1 agonist (D1 ago) and Dopamine D2 antagonist (D2 anta)” or “saline and saline.” Yellow arrowheads show c-fos-positive PV interneurons. The enlargement of c-fos-positive PV interneurons is shown in the square of the lower left. B: Comparison of the c-fos-positive ratio of striatal PV interneurons between cerebellar dystonia mice administered with “D1 ago and D2 anta” and those administered with “saline and saline” (n = 12 in each group). Each point and column represent the mean ± SEM. n.s., not significant, Mann–Whitney *U* test

Abbreviations: PV, parvalbumin; D1 ago, dopamine D1 agonist; D2 anta, dopamine D2 antagonist; DAPI, 4′,6-diamidino-2-phenylindole

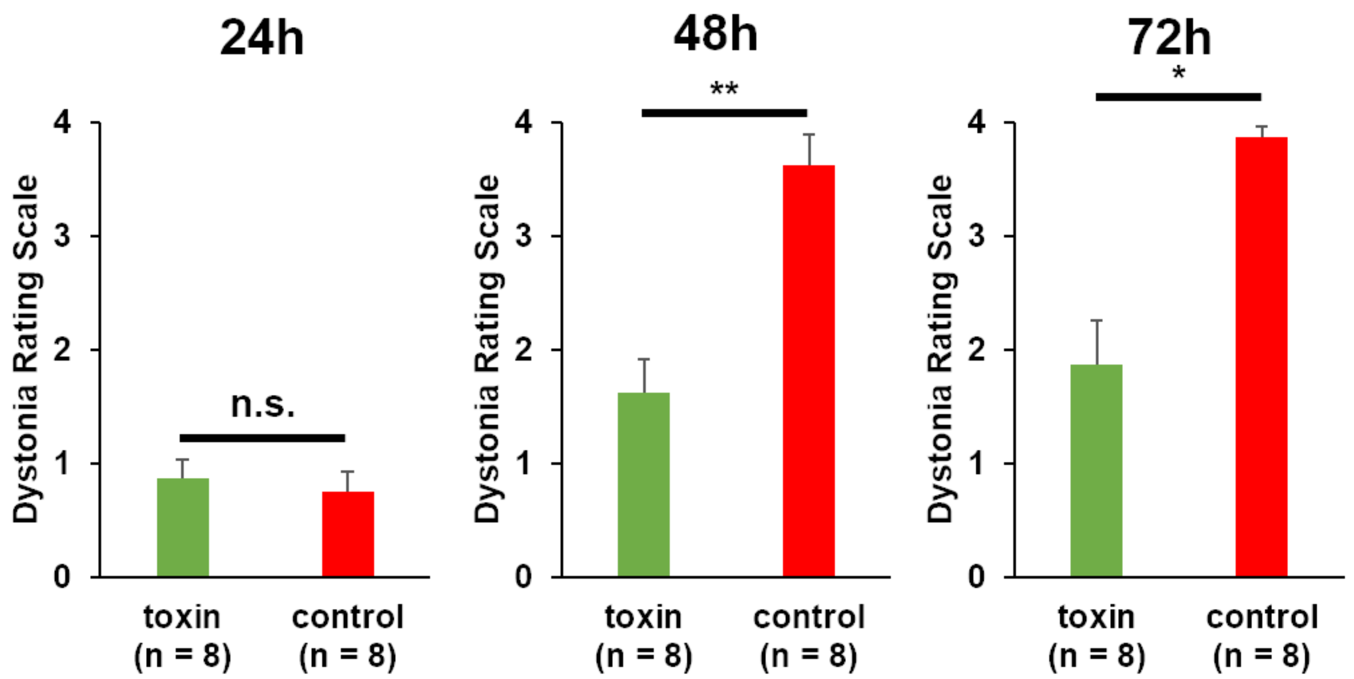

Fig. S8. Changes in the dystonia rating scale of cerebellar dystonia mice treated with second immunotoxin. Comparisons of dystonia rating scale scores at 24, 48, and 72 postoperative hours between cerebellar dystonia mice with selectively ablated dorsolateral parvalbumin interneurons using a second immunotoxin and control (n = 8 in each group).

*p <0.05; **p < 0.01; n.s., not significant; Mann–Whitney *U* test

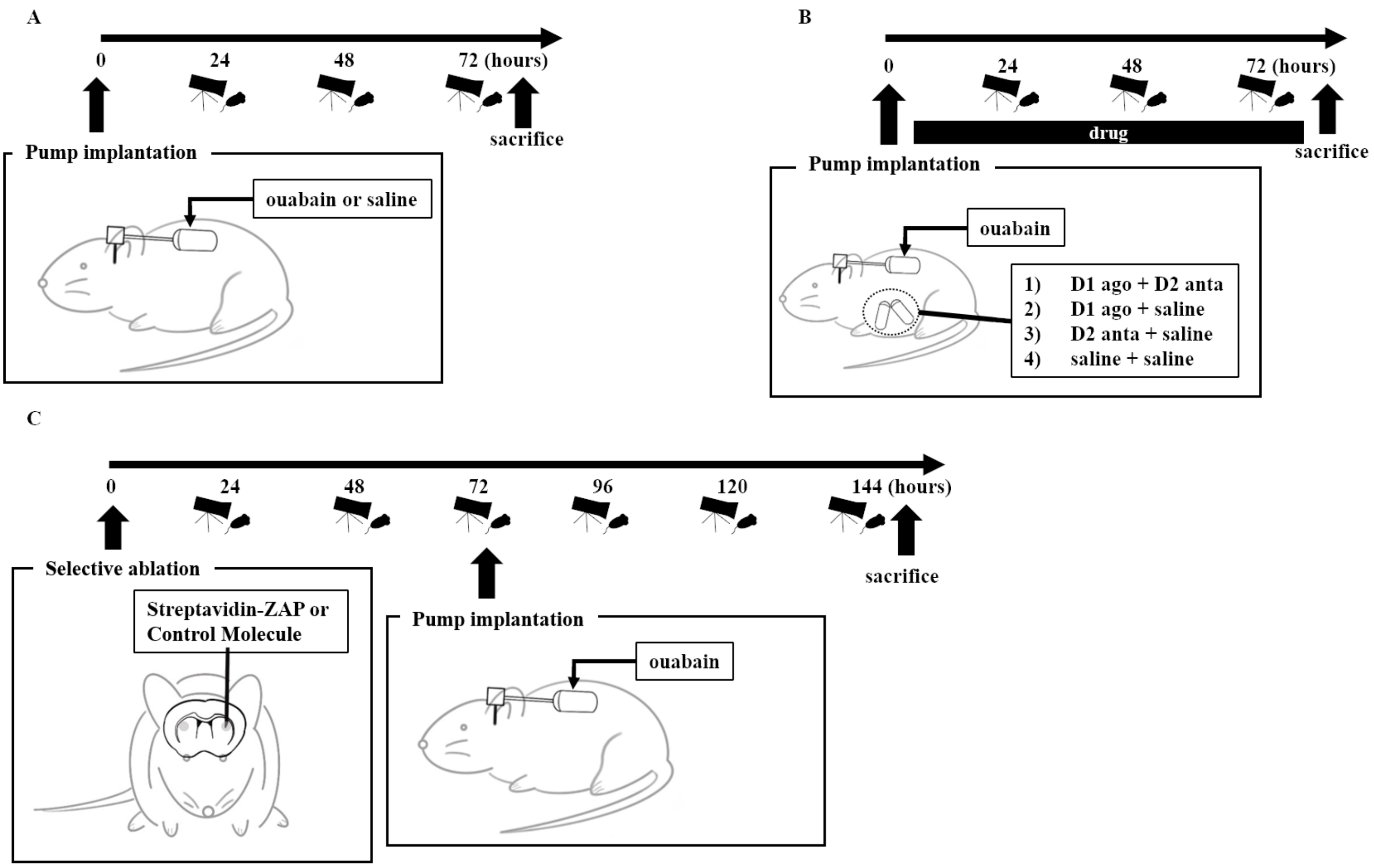

Fig. S9. Time course of the methodology

A: Time course for establishing the mouse model of cerebellar dystonia and the schema of the cerebellar dystonia mouse. B: Time course of drug delivery to the cerebellar dystonia mice and schema of operated mice. C: Time course of selective ablation of PV interneurons to the cerebellar dystonia mice and schema of operated mice

Abbreviations: D1 ago, dopamine D1 agonist; D2 anta, dopamine D2 antagonist; PV, parvalbumin

Disease Models & Mechanisms • Supplementary information

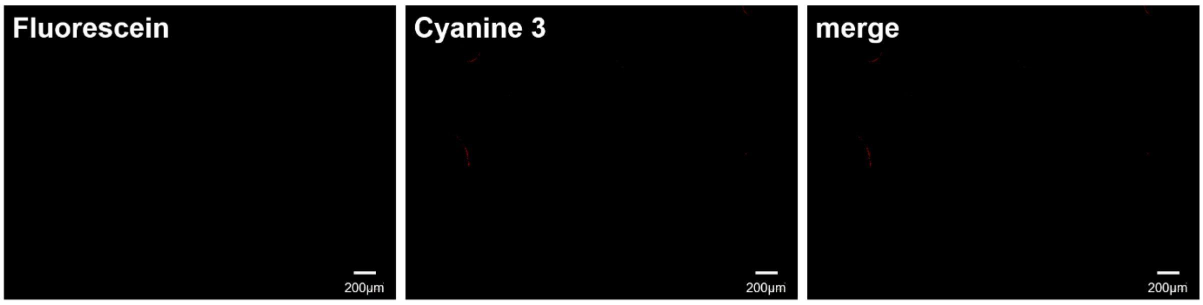

Fig. S10. Negative control of immunohistochemistry

Negative control of immunohistochemistry was detected using fluorescein, cyanine 3, and their merge.

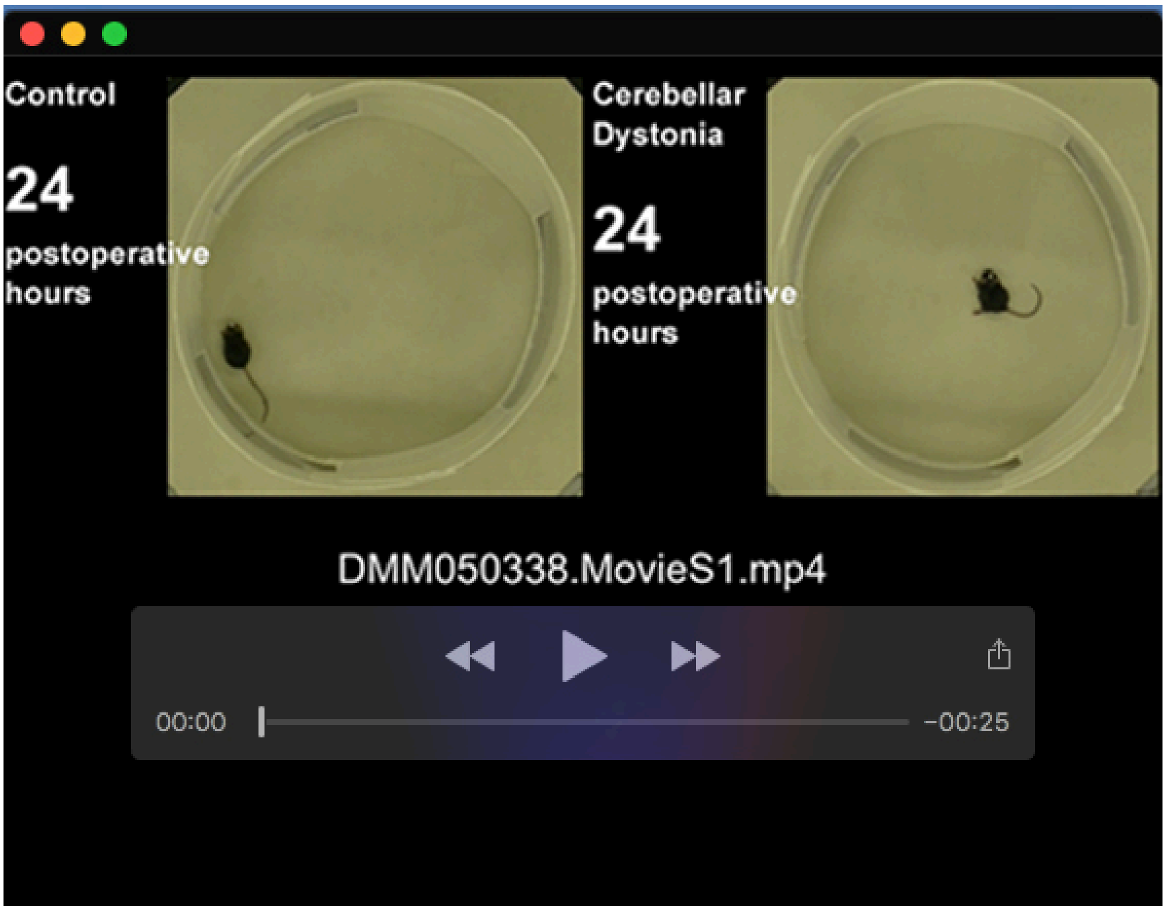

Movie 1. Movies of cerebellar dystonia and control mice.

The behaviors of cerebellar dystonia and control mice at 24, 48, and 72 postoperative hours.

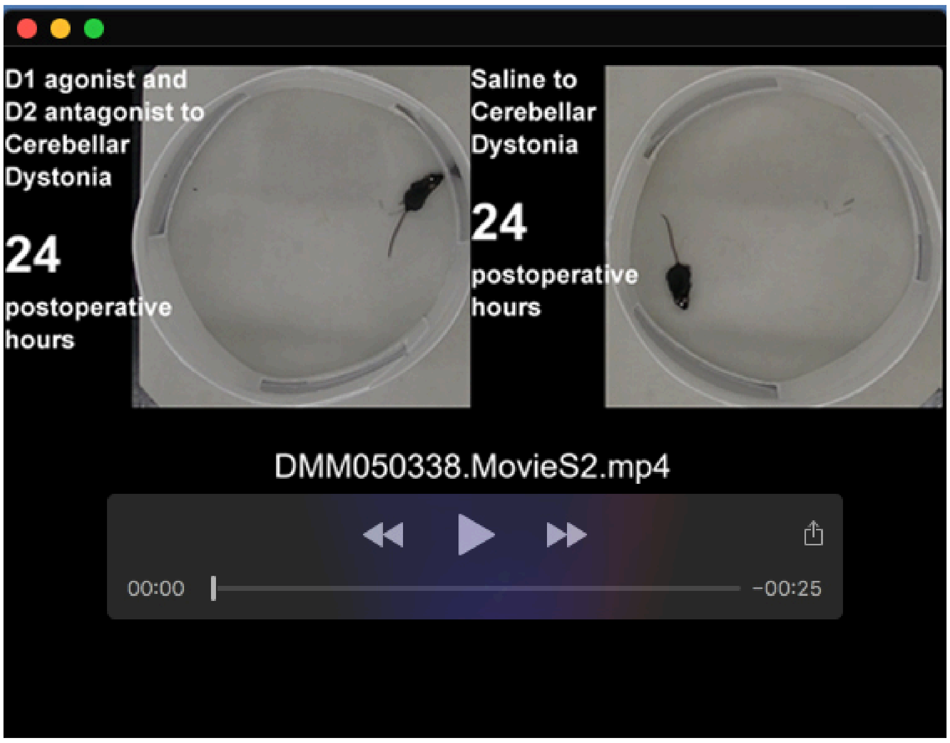

Movie 2. Movies of cerebellar dystonia mice treated with D1 agonist and D2 antagonist

The behaviors of cerebellar dystonia mice that were administered “dopamine (D) 1 agonists and D2 antagonists” and “saline and saline.”

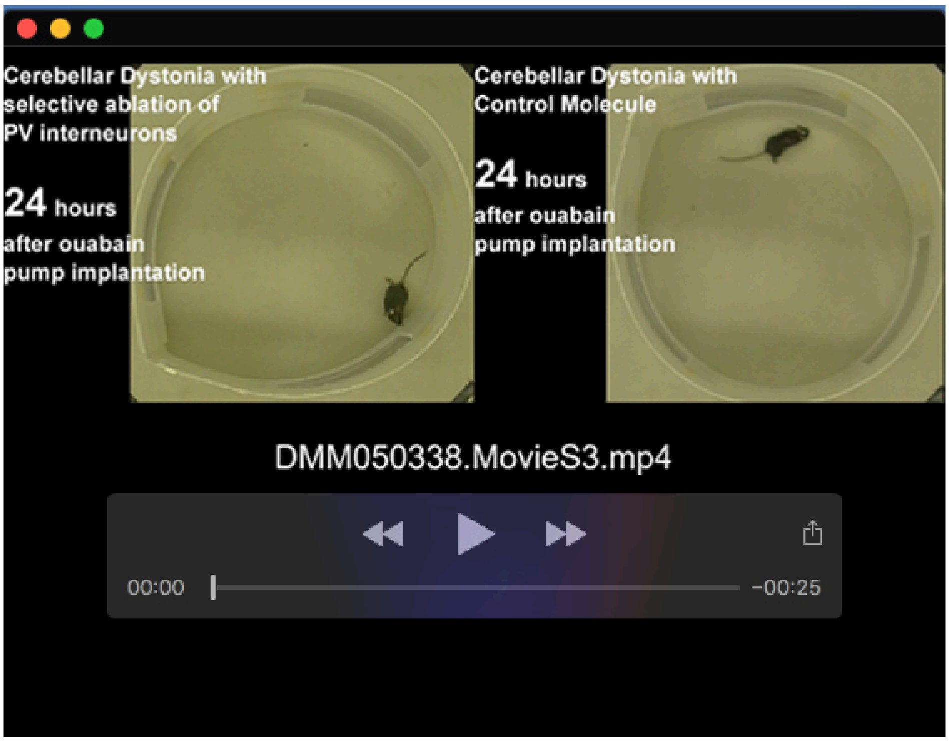

Movie 3. Movies of cerebellar dystonia mice treated with a second immunotoxin

The behaviors of cerebellar dystonia mice with selectively ablated dorsolateral parvalbumin interneurons using second immunotoxin and control at 24, 48, and 72 postoperative hours.

Disease Models & Mechanisms • Supplementary information

Table S1. Characteristics of the primary antibodies

Antigen	Source	Catalog Number	RRID	Clone	Animal	Dilution
c-fos	Millipore	PC38	AB_2106755	Polyclonal	Rabbit	1:50,000
Tyrosine hydroxylase	Abcam	ab137869	AB_2801410	Monoclonal	Rabbit	1:50,000
DARPP-32	Abcam	ab40801	AB_731843	Monoclonal	Rabbit	1:500,000
NeuN	Abcam	Ab177487	AB_2532109	Monoclonal	Rabbit	1:500,000
Methionine enkephalin	Enzo	BML-EA1150-0100	AB_2051851	Polyclonal	Rabbit	1:5,000
Substance P	Abcam	ab67006	AB_1143173	Polyclonal	Rabbit	1:10,000
Parvalbumin	Abcam	ab181086	AB_2924658	Monoclonal	Rabbit	1:50,000
Choline acetyltransferase	Abcam	ab178850	AB_2721842	Monoclonal	Rabbit	1:100,000
FoxP2	Abcam	ab16046	AB_2107107	Polyclonal	Rabbit	1:500,000

Abbreviations: DARPP-32, dopamine and cyclic adenosine monophosphate-regulated phosphoprotein 32 kDa

Disease Models & Mechanisms • Supplementary information
